# Imperfect DNA mirror repeats in the *gag *gene of HIV-1 (HXB2) identify key functional domains and coincide with protein structural elements in each of the mature proteins

**DOI:** 10.1186/1743-422X-4-113

**Published:** 2007-10-26

**Authors:** Dorothy M Lang

**Affiliations:** 1 School of Contemporary Sciences, University of Abertay-Dundee, Bell Street, Dundee DD1 1HG, Scotland, UK

## Abstract

**Background:**

A DNA mirror repeat is a sequence segment delimited on the basis of its containing a center of symmetry on a single strand, e.g. 5'-GCATGGTACG-3'. It is most frequently described in association with a functionally significant site in a genomic sequence, and its occurrence is regarded as noteworthy, if not unusual. However, imperfect mirror repeats (IMRs) having ≥ 50% symmetry are common in the protein coding DNA of monomeric proteins and their distribution has been found to coincide with protein structural elements – helices, β sheets and turns. In this study, the distribution of IMRs is evaluated in a polyprotein – to determine whether IMRs may be related to the position or order of protein cleavage or other hierarchal aspects of protein function. The gag gene of HIV-1 [GenBank:K03455] was selected for the study because its protein motifs and structural components are well documented.

**Results:**

There is a highly specific relationship between IMRs and structural and functional aspects of the Gag polyprotein. The five longest IMRs in the polyprotein translate a key functional segment in each of the five cleavage products. Throughout the protein, IMRs coincide with functionally significant segments of the protein. A detailed annotation of the protein, which combines structural, functional and IMR data illustrates these associations. There is a significant statistical correlation between the ends of IMRs and the ends of PSEs in each of the mature proteins. Weakly symmetric IMRs (≥ 33%) are related to cleavage positions and processes.

**Conclusion:**

The frequency and distribution of IMRs in HIV-1 Gag indicates that DNA symmetry is a fundamental property of protein coding DNA and that different levels of symmetry are associated with different functional aspects of the gene and its protein. The interaction between IMRs and protein structure and function is precise and interwoven over the entire length of the polyprotein. The distribution of IMRs and their relationship to structural and functional motifs in the protein that they translate, suggest that DNA-driven processes, including the selection of mirror repeats, may be a constraining factor in molecular evolution.

## Background

A DNA mirror repeat is a sequence segment delimited on the basis of its containing a center of symmetry on a single strand and identical terminal nucleotides. For example, in the sequence below, TACACG is the mirror image of GCACAT.

    <----------  ---------->

5'- T A C A C G  G C A C A T -3'

3'- A T G T G C  C G T G T A -5'

Imperfect DNA mirror repeats (IMRs) are less than 100% symmetrical.

The identification of mirror repeats is highly dependent on how they are defined. One method is to identify all mirror repeats within a sequence by systematically evaluating the symmetry of each string within in it. This method identifies relatively long (or maximal) symmetric strings (mIMRs). Using symmetry criteria of ≥ 50% and discounting strings completely contained within other strings, the longest mIMRs in TnsA were found to coincide with key structural domains [1].

Another type of mirror repeat is identified by progressively evaluating, from the start to the end of a sequence, symmetric sub-strings bounded by reverse dinucleotides (rdIMRs). These are generally shorter than and often contained within mIMRs. Lang [1] found statistically significant correlations for the coincidence of the ends of rdIMRs and the ends of protein structural elements – helices, β-sheets and turns – in 17 monomeric proteins. In TnsA (*E. coli*), 88% of the known or potential functional motifs occur within rdIMRs and the longest mIMRs translate key functional and/or structural sequences of the protein.

In this study, the distribution of IMRs is evaluated in a gene that translates a polyprotein. The specific goals were to determine whether IMRs span the entire polyprotein, to identify the relationship of IMRs in the precursor to IMRs in the mature cleavage products and to assess the relationship between IMRs and protein functional and structural motifs. The HIV-1 gag sequence used for this analysis is HXB2_LAI_IIIB_BRU [Genbank: K03455], the most commonly used reference sequence for the HIV-1 genome [2]. The *gag *gene of HIV-1 is about twice as long as *TnsA*, and translates the following proteins (in the order of their occurrence within the sequence): matrix (MA), capsid (CA), p2 (SP1), nucleocapsid (NC), and either (a) p1 (SP2) and p6 or (b) GagTF. CA is about the same length as TnsA. The cleavage positions for each of the mature proteins of Gag (HXB2) are summarized in Table 1.

**Table 1:** Nucleotide and amino acid sequences adjacent to cleavage sites in Gag (HXB2) [2]

Segment		DNA	Amino Acid
	nt	start..stop	start..stop
*gag *thru slip	1296	1-atgggtgcg..gctaat-1296	1-MGARAS..ERQAN-432
matrix	396	1-atgggtgcg..aattac-0396	1-MGARAS..VSQNY-132
capsid	693	397-cctata..gttttg-1089	133-PIVQN..KARVL-363
p2	42	1090-gctgaa..ataatg-1131	364-AEAMS..SATIM-377
p7 nucleocapsid	162	1132-atgcag..gctaat-1296	378-MQRGN..ERQAN-432
p1 start is slip	48	1297-ttttta..aatttt-1344	433-FLGKI..RPGNF-448
p6	159	1345-cttcag..caataa-1503	449-LQSRP..DPSSQ$-501
			
*gag-pol TF*	165	1299-tttagg..aacttc-1463	433-FREDL..VSFNF-488

Gag proteins are the structural components of the HIV-1 virus and cleavage of the Gag polyprotein into several mature proteins is essential to replication. Near the C-terminal of Gag (at the NC-p1 cleavage site), the protein becomes polycistronic. The ribosome "slips" within the DNA motif "tttttt", once in every 20^th ^Gag transcription and the resulting transcript is GagTF-Pol. At maturation, the Pol segment is cleaved into enzymatic proteins. Gag and Gag-Pol are cleaved differentially and in stages. This process is summarized in Table 2.

**Table 2:** Gag and Gag-Pol are differentially cleaved at maturation

Gag	stage 1	MA-CA-p2\1/NC-p1-p6
Gag-Pol	stage 1	MA-CA-p2\1/NC-GagTF-pol
		
Gag	stage 2	MA\2/CA-p2\1/NC-p1\2/p6
Gag-Pol	stage 2	MA\2/CA-p2\1/NC\2/GagTF-Pol
		
Gag	stage 3	MA\2/CA\3/p2\1/NC\3/p1\2/p6
Gag-Pol	stage 3	MA\2/CA\3/p2\1/NC\2/PR\3/RT\3/RNase\3/IN

GagTF-Pol results from a frame shift at the end of NC. In Gag, p1 is not cleaved from NC until stage 3. GagTF is cleaved from NC at stage 2 [3-7].

In order to facilitate the comparison of multiple types of data within the context of the protein, a comprehensive annotation of complete Gag sequence was made (Additional file 1) that combines experimentally determined functional and structural motifs, and the sequence positions of IMRs found in this study.

## Results

The five longest mIMRs in *gag *that are ≥ 50% symmetric each translate an essential protein motif in a different cleavage product, indicating that the association between mIMR length and function may be related to selection in both the polyprotein and its cleaved products. Most IMRs translate distinct, functionally significant protein motifs. At symmetry ≥ 50% there are significant statistical correlations between the ends of both mIMRs and rdIMRs, and the ends of protein structural elements (PSEs). Several mIMRs that are ≥33% symmetric start or stop at cleavage positions.

The DNA and amino acid sequence positions of the longest L1 mIMRs are listed in Table 3. The designation L1 means that it is the longest IMR for a unique span of the DNA sequence. MIMRs are identified by evaluating the symmetry of every possible sub-string of a DNA sequence, then nesting them sequentially, beginning at the 5' end. The span of the first IMR is designated L1; all shorter IMRs within the span are designated progressively higher levels (L2, L3, etc.) based on whether they are completely contained within another IMR. The next L1 IMR ends downstream from the end of the preceding IMR; it may begin within a preceding IMR or downstream from it. For the remainder of this article, all references to IMRs refer to L1 IMRs. Each (L1) mIMR is assigned an ID number based on rank by length, and is preceded by a hash mark (e.g. #1-gag). The position of some mIMRs differ by only a few amino acids, so it is possible to simplify the data by discounting mIMRs that substantially overlap. Table 4 summarizes this simplification and illustrates that although mIMRs occur throughout most of the Gag protein each span is associated with distinct structural or functional domains.

**Table 3:** mIMRs in gag that are ≥50% symmetrical

**Rank**	**m-IMR ID**	**protein**	**length**	**DNA positions**	**protein positions**	**overlaps**
1	#1-gag	MA	95	0270-aa..ca-0364	091-RI..DT-122	
2	#2-gag	CA	87	0742-gg..tg-0828	248-GW..RM-276	
3	#3-gag	NC-p1-p6	85	**1256-aa..aa-1340**	419-EG..GN-447	
	#4-gag	MA	82	0266-at..ca-0347	089-HQ..AQ-116	~#1-gag
	#5-gag	CA	81	**0758-at..ta-0838**	253-NP..PT-280	~#2-gag
4	#6-gag	NC	81	1171-aa..ga-1251	391-KC..CG-417	
5	#7-gag	p2	80	**1065-ac..ca-1144**	356-PG..GN-382	
	#8-gag	CA	77	0764-ct..cc-0840	255-PP..PT-280	~#2-gag
	#9-gag	gag	77	**1100-tg..gt-1176**	367-MS..KC-392	~#7-gag
6	#10-gag	CA	76	**0812-at..ta-0887**	271-NK..DY-296	
7	#11-gag	CA	75	**0920-ag..ga-0994**	307-EQ..KT-332	
	#12-gag	MA	74	0299-ct..ac-0372	100-AL..GH-124	~#1-gag
	#13-gag	MA	71	0303-ag..ca-0373	102-DK..HS-125	~#1-gag
8	#14-gag	CA	69	**0985-ga..ag-1053**	329-DC..QG-351	
9	#15-gag	CA	64	0543-ca..aa-0606	181-PQ..LK-202	
	#16-gag	CA	64	**0810-aa..aa-0873**	271-NK..KE-291	~#10-gag
10	#17-gag	p6	64	1362-gc..ag-1425	455-PT..QK-475	
	#18-gag	MA	63	**0265-ca..ac-0327**	089-HQ..QN-109	~#1-gag
11	#1-NC	NC	59	**1209-aa..aa-1267**	404-NC..QM-423	
12	#2-NC	NC	47	1153-aa..ca-1199	385-NQ..GH-400	

ID numbers for each mIMR (e.g. #1-gag) are based on rank by length (#1 being the longest). MIMRs terminated by reverse dinucleotides are bold.

**Table 4:** Simplification of Table 3 by removal of slightly overlapping mIMRs

**Rank**	**mIMR**	**prot**	**len**	**DNA positions**	**AA positions**	**Structure or function**
1	#1-gag	MA	95	0270-aa..ca-0364	091-RI..DT-122	MA-H5 related to viral entry
2	#2-gag	CA	87	0742-gg..tg-0828	248-GW..RM-276	CA-H7 longest constituent of viral core
3	#3-gag	p1	85	1256-aa..aa-1340	419-EG..GN-447	end NC, p1 to p1-p6 cleavage site
4	#6-gag	NC	81	1171-aa..ga-1251	391-KC..CG-417	1st cys-his box; EF1α binding
5	#7-gag	p2	80	1065-ac..ca-1144	356-PG..GN-382	p2, critical to budding
6	#10-gag	CA	76	0812-at..ta-0887	271-NK..DY-296	major homology region
7	#11-gag	CA	75	0920-ag..ga-0994	307-EQ..KT-332	endocytosis signal 1; CA-H9 helix
8	#14-gag	CA	69	0985-ga..ag-1053	329-DC..QG-351	endocytosis signal 2; CA-H10 helix
9	#15-gag	CA	64	0543-ca..aa-0606	181-PQ..LK-202	CA-H3-H4 helices, part of viral core
10	#17-gag	NC	64	1362-gc..ag-1425	455-PT..QK-475	L-domain (budding); Tsg101
					455-PT..QK-475	docking; ubiquitin-gag conjugate
						
11	#1-NC	NC	59	1209-aa..aa-1267	404-NC..QM-423	2cd cys-his box; end NC
12	#2-NC	NC	47	1153-aa..ca-1199	385-NQ..GH-400	EF1α binding

MIMRs that begin and end within two amino acids of a larger mIMR have been removed. Although the distribution of mIMRs is nearly continuous throughout gag, the functional and/or structural association of each is discrete, as indicated by the structure-function notation in the right hand column of this table, which is described in greater detail in Additional File 1.

MIMRs were found separately for the Gag polyprotein and each of the cleavage products. It was anticipated that the mIMRs for Gag CDS would be different than those for the components, but they were not except that there are two mIMRs in the NC that only attain L1 status when NC is evaluated separately (not as part of *gag*). The distribution of mIMRs in Gag indicates that most of the largest mIMRs do not span sequences that will be cleaved into separate proteins. The single exception is E419..E454 (#3-gag), which spans NC-p1, and terminates at the p1-p6 cleavage site; this is the segment that is differentially cleaved in Gag and Gag-Pol.

Table 5 lists the DNA and amino acid sequence positions of the longest rdIMRs. RdIMRs are identified by sequentially evaluating, from 5' to 3', the symmetry of each substring delineated by each dinucleotide and the next downstream reverse dinucleotide. They are nested by the same process described for mIMRs. Most of the protein segments translated by rdIMRs coincide with experimentally determined structural or functional motifs of the protein.

**Table 5:** rdIMRs in gag ranked by length

**Beg**		**end**	**rd-IMRs**	**nt**	**prot**	**AA**	**structure or function**
1215	ca .. ac	1261	$1-gag	47	NC	R406..H421	primer annealing; Cys-His box
767	tc .. ct	803	$2-gag	37	CA	I256..L268	N-terminal CA-H7 helix
73	gg .. gg	108	$3-gag	36	MA	G025..W036	nuclear localization signal 1(NLS1)
1245	at .. ta	1279	$4-gag	35	NC	C416..T427	Zn finger motifs. 2cd cys-his box
267	tc .. ct	300	$5-gag	34	MA	Q090..A100	I92..V95 affect struct orientation of MA-H5
168	ct .. tc	200	$6-gag	33	MA	C057..S067	MA H3 helix, C57S prevents particle fmtn
68	ca .. ac	97	$7-gag	30	MA	P023..H033	basic residues target, bind Gag to PM
1379	aa .. aa	1408	$8-gag	30	p6	E460..T470	possible association with ubiquitin
184	gg .. gg	212	$9-gag	29	MA	G062..G071	C-terminal MA-H3 helix
198	at .. ta	226	$10-gag	29	MA	P066..R076	essential to structural transformation
246	ag .. ga	274	$11-gag	29	MA	A083..I092	mutations retarget assembly
232	tt .. tt	259	$12-gag	28	MA	L078..C087	MA-H4 central to 3D structure
1091	ct .. tc	1118	$13-gag	28	-	A364..S373	cleavage site, most of p2
618	tg .. gt	644	$14-gag	27	CA	E207..V215	C-terminal CA-H4 helix, CypA interaction
1074	ta .. at	1100	$15-gag	27	-	K359..M367	cleavage CA-p2
490	tt .. tt	515	$16-gag	26	CA	F164..F172	folds against MHR
1108	ag .. ga	1131	$17-gag	24	-	V370..M378	cleavage site p2-p7, phosphorylation
385	ag .. ga	409	$18-gag	25	-	S129..N137	spans cleavage site MA-CA
894	cc .. cc	918	$19-gag	25	CA	R299..A306	end MHR
964	tt .. tt	988	$20-gag	25	CA	L322..C330	interacts with LysRS
1027	ct .. tc	1051	$21-gag	25	CA	L343..Q351	interacts with LysRS
650	ca .. ac	673	$22-gag	24	CA	P217..P225	CypA interaction; surface of virion core
1043	ca .. ac	1066	$23-gag	24	CA	T348..P356	interacts with LysRS
590	cc .. cc	612	$24-gag	23	CA	A197..T204	N-terminal CA-H4 helix
931	ca .. ac	953	$25-gag	23	CA	Q311..T318	N-terminal CA-H9 helix, LysRS interaction
1275	tt .. tt	1297	$26-gag	23	NC	C426..F433	cleavage site p7-p1
12	ag .. ga	33	$27-gag	22	MA	A005..G011	links myristoylation and calmodulin-binding
712	gg .. gg	733	$28-gag	22	CA	G238..E245	links CA-H5 and -H6 helices
1109	ta .. at	1130	$1-p2	22		V370..M377	cleavage site p2-p7, phosphorylation
1	at .. ta	21	$1-MA	21		M001..V007	minimum signal required for myristoylation
135	ag .. ga	155	$2-MA	21		V046..S052	tether btwn MA at viral membrane and CA
150	gt .. tg	170	$3-MA	21		L051..C057	L50A-L51A prevents particle formation
109	gc .. cg	128	$4-MA	20		A037..R043	binds HIV to plasma membrane, calmodulin
327	ca .. ac	346	$5-MA	20		K110..Q116	nuclear localization signal 2
45	at .. ta	63	$6-MA	19		W016..L021	mutations retarget particle fmtn to Golgi
166	gg .. gg	184	$7-MA	19		G056..G062	G56E, C57D, C57S, I60E elim replication
95	aa .. aa	112	$8-MA	18		K032..S038	binds HIV to plasma membrane, calmodulin
139	aa .. aa	156	$9-MA	18		N047..E052	?
320	ag .. ga	337	$10-MA	18		E107..K113	nuclear localization signal 2
22	tt .. tt	38	$11-MA	17		L008..L013	?
367	gg .. gg	382	$12-MA	16		G123..V128	start labile structure near cleavage site
565	aa .. aa	585	$1-CA	21		N189..Q195	connects CA-H3 and CA-H4 helices
647	at .. ta	667	$2-CA	21		H216..I223	CypA interaction; surface of virion core
676	gg .. gg	696	$3-CA	21		G226..R232	CypA interaction; surface of virion core
947	gg .. gg	967	$4-CA	21		W316..V323	CA H9 helix, endocytosis signal
1039	at .. ta	1059	$5-CA	21		M347..V353	interacts with LysRS
832	ag ga	851	$6-CA	20		S278..D284	necessary for formation of dimer interface
910	ct .. tc	929	$7-CA	20		L304..S310	spans CA-H8-H9 helices
927	tt .. tt	946	$8-CA	20		S310..W316	N-terminal, LysRS interaction site
1306	aa .. aa	1325	$1-p1	20		K436..K442	start is slip site, p1 protein
1319	cc .. cc	1334	$2-p1	16		S440..P445	middle, p1 protein

The rank of each rdIMR within the entire gag gene was determined first, then rank within each mature protein. Multiple rdIMRs of the same length were ordered by sequence position.

**Figure 1:**
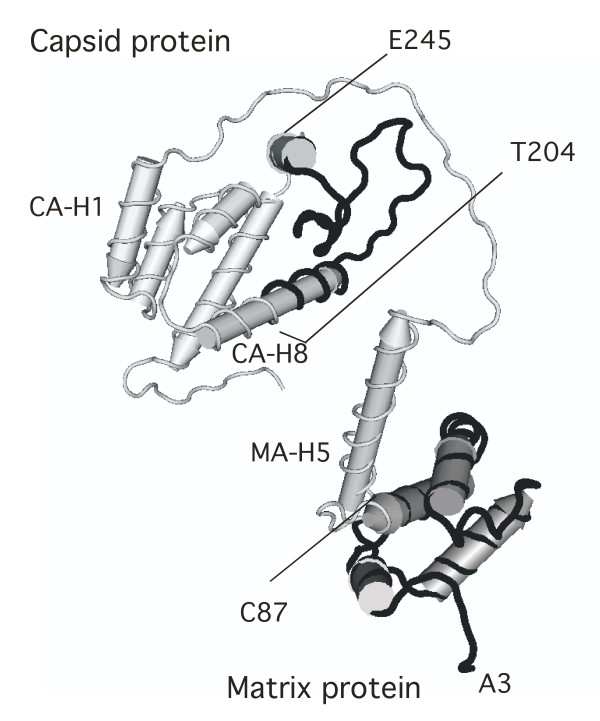
**The distribution of mIMRs in the immature Gag protein [NCBI:1L6N, [8]]**. MIMRs that are ≥ 50% symmetric are noticeably absent from some segments of the protein. These regions are characterized by a series of rdIMRs, arranged end-to-end (illustrated in black). The spans lacking mIMRs are highly reactive and mobile. The A3..C87 region of matrix undergoes structural transformation at several stages of the virion life cycle, and contains basic residues that target Gag to the plasma membrane [9], a calmodulin-binding motif [10] and a nuclear localization signal [11]. The T204..E245 region of capsid includes the exposed loop on the virion core [8, 12], and the *CypA *binding site [12].

MIMRs and rdIMRs vary in distribution, beyond that which would occur due to the differences in their lengths. MIMRs occur throughout most of *gag*, as a series of overlapping, or nearly overlapping spans; within many mIMRs, there are one or two spatially separated rdIMRs. MIMRs are, however, noticeably absent in some segments of *gag*; in these segments, e.g. M1..R91 (MA) and P133..G248 (CA), rdIMRs form a nearly continuous series, end-to-end. The sequence spans in MA and CA that do not contain mIMRs are illustrated in Figure 1. These regions are both highly reactive and mobile (detailed in the legend).

**Figure 2:**
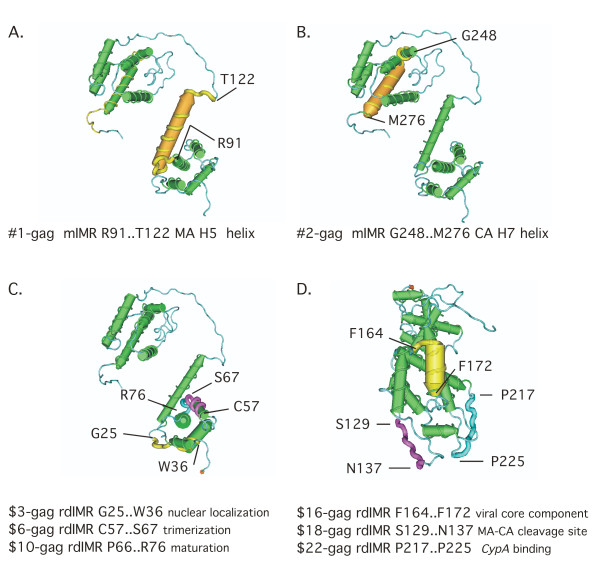
**The longest IMRs coincide with key protein functional motifs**. Figures **2A **and **2B [NCBI:1L6N [8]] **illustrate the two longest mIMRs in the Gag polyprotein – #1-gag in matrix and #2-gag in capsid. These mIMRs translate the MA H5 and CA H7 helices which (in the illustrated structure) are approximately parallel to each other at a pitch of about 45°. Both are essential to the structure and function of each protein. Figure **2C **illustrates the largest rdIMRs in matrix and Figure **2D **the largest rdIMRs in capsid, that do not coincide with mIMRs.

Figures 2A and 2B illustrate the protein translation of the two largest mIMRs in *gag *– the largest helix in MA (2A) and CA (2B) and the adjacent turns essential to the tertiary structure. The PDB structure used for this illustration – 1L6N – is of the immature Gag protein; the structure of MA and CA is not substantially different in the mature proteins, except that the long loop between them is cut and refolded [8]. The MA-H5 helix is distinct from the other matrix components, and in the mature protein projects directly into the center of the virion [13]; the MA-H5 helix may also contain a nuclear localization signal [11]. The CA-H7 helix stabilizes interface 1 (planar strips) of the viral core [14].

Figures 2C and 2D illustrate the three largest rdIMRs in MA and CA. The protein translation of $3-gag spans a nuclear localization signal; $6-gag and $10-gag are essential to structural transformation at maturation [15]. The protein translation of $16-gag spans a region that refolds to create a CA-CA interface essential to assemble the core [16]; $18-gag spans the MA-CA cleavage site; $22-gag translates part of the loop on the surface of the virion core and interacts with *CypA *[12].

**Figure 3:**
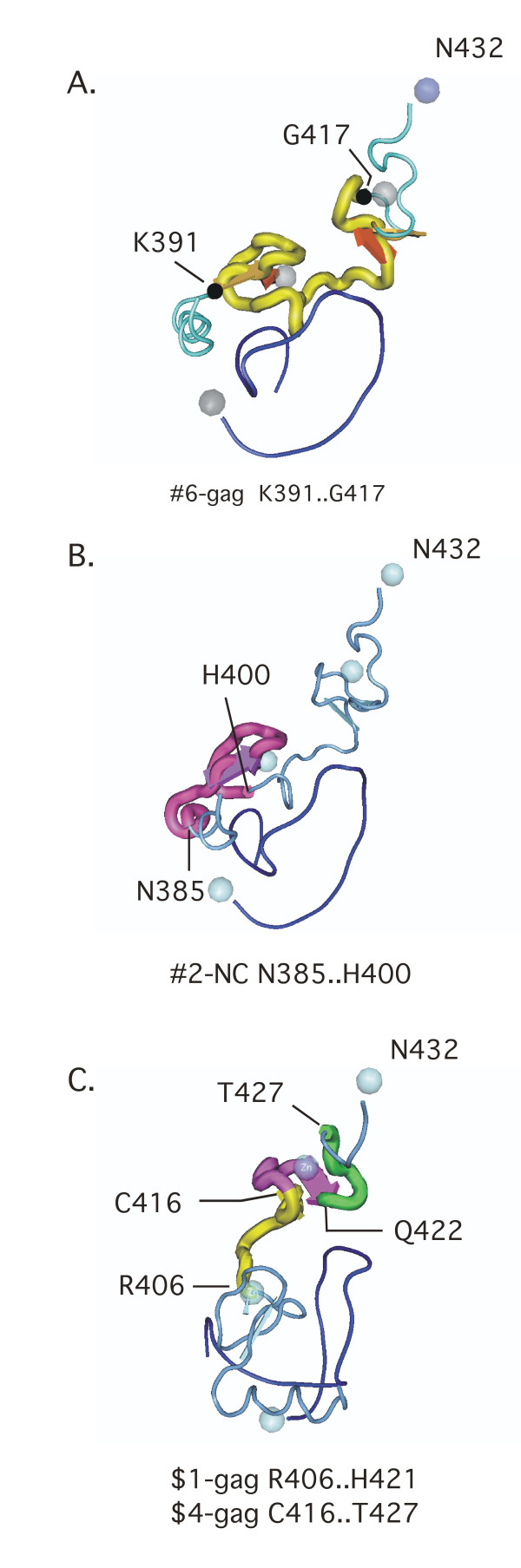
**The largest mIMR in the nucleocapsid spans the two Cys-His boxes [NCBI:1F6U [18]]**. Figure **3A **illustrates the largest mIMR in the nucleocapsid – #6-gag. This mIMR spans both zinc knuckles and the spacer between them. Each of the next largest mIMRs in the NC, translates one of the Cys-His boxes. Figure **3B **illustrates the first Cys-His box. Figure **C **(same polar orientation as A and B, but rotated) illustrates the two longest rdIMRs in Gag that occur in the nucleocapsid – $1-gag and $4-gag – which overlap; within the overlap region (in purple) two amino acids bind the zinc ion [19].

Figure 3 illustrates the two largest mIMRs in the nucleocapsid. The largest (Fig. 3A) spans the entire region connecting the two Cys-His boxes. The second largest (Fig. 3B) spans the EF1α binding site and first Cys-His box. The largest rdIMRs in the NC overlap (Fig. 3C), and a Zn ion is bound within the region translated by the overlap. The Cys-His boxes are zinc finger binding domains which enable NC to bind to nucleic acids, and the Zn ion increases the affinity of NC for nucleic acids; NC also has unwinding properties, resembling a DNA topoimerase [17].

The coincidence of the ends of IMRs and PSEs was tested for several gene segments – MA-CA-p2-NC, MA, CA and NC segments – using Fisher's exact test (FET) [20]. The Kabsch and Sander [21] secondary structure prediction was used with the 1L6N tertiary structure (PDB) and statistically significant values were found for the MA-CA-p2-NC, CA and NC segments; PROMOTIF secondary structure annotation was used for MA. These results are summarized in Table 6.

**Table 6:** Both mIMRs and rdIMRs coincide with PSEs in each mature protein and the polyprotein

**DNA segment**	**MIMRs**			**mIMRs terminated by reverse dinucleotides**			**rdIMRs**			
	N*	max	FET	N*	max	FET	N*	length	max	FET
			p-value			p-value				p-value

MA-CA-p2-NC	4337	-7	0.0513	2141	-7	0.0190	2529	all	4	0.0163
MA-CA-p2-NC							1267	≥ 16 nt	5	0.0526
										
MA-CA-p2	3907	-8	0.0084	2045	-7	0.0085	2196	all		none
MA-CA-p2							1302	≥ 15 nt	-5	0.0356
										
MA – K&S	1463	-8	0.0088	746	-8	0.0034				none
MA – promotif										none
MA – promotif							502	≥ 15 nt	3	0.0154
										
CA	2364	7	0.0103	1312	6	0.0409	1354	all	4	0.0757
CA							637	≥ 16 nt	-2	0.0019
										
NC	421	-2	0.0004	144	-1	0.0110	334	all	1	0.0019
NC							149	≥ 16 nt	7	0.0422
NC							114	≥ 19 nt	7	0.0027

The coincidence of IMRs and PSEs was tested for each of the sequentially cleaved segments, and found to be valid for all of them. For most segments, the correlation is improved when short IMRs below the essential value are removed, indicating that the coincidence is related to sequence segments longer than 15 nt.

The mIMRs included in the test are all ≥58 nt and often span more than a single protein structural element. The rdIMRs included in the test are all ≥15 nt. Both mIMRs and rdIMRs begin and end at various positions within codons and therefore, the composition of the two nucleotides at each end (which delimit the rdIMRs) are unlikely to be strongly influenced by preferences related to secondary structure composition or codon preference. More than 50% of the mIMRs are terminated by reverse dinucleotides.

For almost all measurements of coincidence, the ends of IMRs and PSEs were statistically significant over a range of 3 nt, similar to the span found in TnsA. The position at which the coincidence is maximal is listed in Table 6. The coincidence of IMR and PSE at position 0 indicates that the span of a PSE exactly coincides with the span of an IMR. When the position is negative, the IMR begins slightly upstream of the start of the PSE; when the position is positive, the IMR begins slightly downstream. The difference is indicated as a nucleotide position, however, so in the protein the equivalent distance is 1–2 amino acids, which is similar to the variability of different structure prediction methods.

Differences in the position of maximum coincidence between the segments occur for several reasons. The measurement includes coincidences over the entire range of the sequence, and the position of maximum coincidence would be expected to be somewhat different for each protein due to differences in secondary and tertiary structure. The values, however, are consistent; the largest segment – MA-CA-p2-NC – has a maximum coincidence at position 5 (for rdIMR ≥16 nt), which is central to positions 3, -2 and 7, which are maximal for MA, CA and NC, respectively.

The coincidence of IMRs with PSEs may be enhanced by the greater than expected numbers of them in the Gag polyprotein. The following formula predicts the expected number of occurrences.

P(t) predicted number of occurrences of mIMRs in the sequence

P(o) probability of the occurrence of a mirror repeat in a random sequence consisting of 4 nucleotides present in approximately equal amounts

P(e) probability of the ends of a segment matching, for mIMRs, P(e) = 1/4

P(m) probability of number of matches required for symmetry

l number of potential matches (1/2 total sequence length, odd values disregarded)

m number of matches required for symmetry

P(o) = P(e) * P(m)

P(m) = (l!/((m!(l-m)!) * (1/4)^m ^* (3/4)^l-m^

In gag, 18 L1 mIMRs were identified that were ≥ 63 nt. Therefore, as a generalization, this length will be evaluated. Since we are only concerned that one side of the segment matches the other, l = 30 and m = 14.

P(m) = (30!/(14! * 14!)) * (1/4)^14 ^* (3/4)^16^

**P(m) = 0.005430**

Adding the criteria that the ends must match,

**P(o) = 0.001357**

The length of gag is 1500 nt, from which is subtracted the required length for the match (62), resulting in 1438 potential sites ≥ 63 nt.

P(t) = P(o) * 1438 = 1.95

This value indicates that it is likely that at least two mIMRs ≥ 63 nt will occur by chance. Since each possible site of an mIMR is included to obtain this estimate, it should be compared with the total number if mIMRs ≥ 63 nt that were identified (= 49), not just L1 mIMRs (= 18). Therefore, the observed frequency (49) is 25-fold greater than the expected frequency (2).

A similar process for rdIMRs can be made, with the only change of P(e) = (1/4)*(1/4), to reflect the reverse dinucleotide criteria delimiter. The estimate will be for rdIMRs ≥20 nt, the length summarized in Table 5.

P(m) = (l!/((m!(l-m)!) * (1/4)^m ^* (3/4)^l-m^

P(m) = (8!/(3! * 5!)) * (1/4)^3 ^* (3/4)^5 ^= 0.2076

P(o) = P(e) * P(m) = (1/16) * 0.2076 = 0.01280

P(t) = P(o) * (1500-19) = 19.2

The observed frequency for rdIMRs ≥20 nt is 53, approximately 2.5 the predicted number.

Both mIMRs and rdIMRs occur at greater than expected numbers, although the greater than expected number of mIMRs is much greater than for rdIMRs. These values demonstrate that it is unlikely that the multiple occurrences of mIMRs ≥63 nt occur by chance. It is also unlikely that chance occurrences will be at positions that are highly significant to the function of the protein.

The affect of modifying symmetry criteria on IMR identity was examined for both lower and higher levels of symmetry. No evidence of a relationship between mIMRs and protein cleavage sites for the entire Gag polyprotein was found at levels of symmetry ≥50%. Table 7 summarizes L1 mIMRs that are ≥33% symmetrical. Using the formula described previously, less than one (0.1128) mIMRs that is 704 nt in length and ≥33% symmetric is expected within the *gag *sequence of 1500 nt; in contrast, five are observed and there are an additional 237 that are longer than 705 nt, indicating that mirror symmetry pervades the gene. About half of the L1 mIMRs translate protein segments that would end at or near cleavage sites, and one mIMR coincides with the start of CA and the end of p6. MIMRs that are not associated with cleavage sites begin and end at functionally related domains.

**Table 7:** MIMRs ≥ 33% begin and end at cleavage sites (bold) and sites that have related functions in the translated protein

**DNA mIMR**	**len**		**protein**		**protein function**
**begin..end**			**begin..end**		
1-atg..tga-704	704	begin	MA,	M-1	start MA
		end	CA,	D-235	CypA binding site – CA uncoating exposed loop virion core surface
9-gag..gag-778	770	begin	MA,	R-4	min myristoylation signal
		end	CA,	E-260	H7, largest component viral core
35-aat..taa-811	777	begin	MA,	E-12	AP-3 binding
					calmodulin binding
					plasma membrane binding
		end	CA,	N-271	H7, largest component viral core
43-cga..tgc-1135	1093	begin	MA,	R-15	AP-3 binding
					calmodulin binding
					plasma membrane binding
		end	p2,	Q-379	**p2-NC cleavage site +1AA stage 1 Gag & Gag-Pol**
153-aga..gga-1228	1076	begin	MA,	E-52	-2AA essential to trimerization & virus assembly
		end	NC,	K-410	motif crucial to NC-RT binding
302-tag..gct-1293	992	begin	MA,	L-101	start, H5 helix related to viral entry
		end	NC,	A-431	**NC-p1 cleavage Gag**
					**NC-GagTF cleavage Gag-Pol**
337-aaa..taa-1459	1123	begin	MA,	K-113	nuclear localization signal
		end	p6,	F-487	**end GagTF**
360-tga..aat-1501	1142	begin	MA,	D-121	end, H5 helix related to viral entry
		end	p6,	$-501	**end Gag (end p6)**
400-ata..taa-1503	1104	begin	MA,	I-134	**MA-CA cleavage + 1AA **Gag & Gag-Pol
		end	p6,	$-501	**end Gag (end p6)**

The region M1..K32 encompasses the start of four mIMRs (≥33% symmetrical) and is the region that targets Gag to the cell membrane [22]. Two of these mIMRs terminate within capsid D235..E260 which is a region of small helices and loops adjacent to the CypA binding site that is probably essential to disassembling the core upon infection [14]; these mIMRs, then, begin at sequences that localize Gag to the cell membrane – a process essential to core formation – and end at sequences that dissolve the virion core (upon infection). Similarly, E12..N271 begins within the membrane localization domain, and ends at CA-H7, the largest component of the structural core, which stabilizes its constituent planar strips [14]. The fourth mIMR, R15..Q379, begins within the membrane localization region and terminates one amino acid downstream from the p2-NC cleavage site; cleavage at p2-NC is the initial step in the Gag cleavage sequence [3]. MIMR E52..K410 begins at positions essential to particle formation, trimerization and virus assembly, and terminates immediately upstream of the second Cys-His box (zinc finger) which is essential to packaging. Several mIMRs begin within the region L101..D121, which includes most of the MA-H5; this helix projects away from the plasma membrane, directly into the center of the virion [23] and deleterious deletions within it have been found to block viral entry [13]. MIMRs that begin at the MA-H5 helix terminate at the NC-p1 cleavage site and the end of Gag-Pol TF and p6. The association of weakly symmetrical mIMRs with cleavage sites in the polyprotein and functionally related protein motifs suggests that different levels of IMR symmetry may be related to different functional aspects of the translated protein.

At higher criteria for symmetry (≥66%), the sequence positions of mIMRs and rdIMRs are nearly the same. These results are summarized in Table 8. At this level of symmetry the distribution of rdIMRs and mIMRs are nearly identical.

**Table 8:** mIMRs and rdIMRs that are ≥66% symmetric

	mIMR DNA		mIMR AA		Protein function	rdIMR AA	
len	begin	end	begin	end		begin	end
37	1158	1194	386	398	1st cys-his box		
37	1314	1350	438	450	p1-p6 cleavage site F449..L450	440	445
36	1087	1122	362	374	p2 helix, most of p2 A364..M377	364	373
36	1349	1384	450	461	L domain P455..P459	448	456
30	74	103	25	34	residues essential to binding to cell membrane	25	36
28	1302	1329	434	443	most of p1 F433..F448	436	442
27	17	43	6	14	myristoylation	8	13
26	562	587	187	196	bridges CA H3 and CA H4 helices	189	195
25	322	346	107	115	part of MA H5 helix T97..A120	107	113
25	478	502	159	167	bridges CA H1 and CA H2 helices		
25	734	758	245	253	bridges CA H6 helix and downstream B-sheet		
25	930	954	310	318	CA H9 helix, endocytosis signal T311..Q324	310	318
25	1022	1046	341	349	CA H11 helix L343..C350	343	351
25	1404	1428	468	476	T471A mutation leads to incomplete separation from host cell membrane		
24	356	379	119	126	labile structure at end of MA	123	128
24	838	861	279	287	required for dimer interface	278	284
24	1146	1169	382	390	g helix, near start p7		
23	772	794	257	265	CA H7, potential NEC cleavage site	256	268
23	1189	1211	396	404	1st cys-his box		
22	1253	1274	418	425	2cd cys-his box	416	427
21	1	21	0	7	minimum signal required for myristoylation	0	7
21	268	288	89	96	loop with highly variable charge btwn MA H4-H5	90	100
21	361	381	120	127	near end of MA	123	128
21	1238	1258	413	419	2cd cys-his box C412..H421	406	421
21	1274	1294	425	431	ends at p7-p1 cleavage site N432..F433	426	433
20	990	1009	330	336	CA H10 helix, endocytosis signal P328..L337	426	433
19	63	81	21	27	basic residues essential to binding P23..H33	23	33
19	159	177	53	59	MA H3 helix, mutations affect virus assembly	51	57
19	192	210	64	70	MA H3 helix, mutations affect virus assembly	62	71
19	305	323	102	108	part of MA H5 helix, potential PDZ domain binding		
19	956	974	319	325	CA H9 helix, endocytosis signal Q311..Q324	316	323
19	1060	1078	353	359	G-rich segment at end CA		
19	1427	1445	476	482	end of Gag-PolTF	476	481
18	468	485	156	162	CA H1 helix		
18	541	558	180	186	CA H3 helix, D184 essential to mature capsid		
18	886	903	295	301	MHR	299	306
18	919	936	306	312	deletion causes major defect in particle formation	304	310
18	956	973	319	324	CA H9 helix, endocytosis signal T311..Q324	316	323
18	1389	1406	463	469	ubiquitin-gag conjugates found L449..Q500	460	470
18	1461	1478	487	493	vpr packaging L489..F493	487	493

Increased stringency for symmetry results in substantial overlap of mIMRs and rdIMRs. Many of the mIMRs listed in this table are relatively short and therefore do not appear in Tables 3, 4 or 5.

## Discussion

In this study, IMRs were found occur in *gag *in greater than expected numbers, and in a hierarchal order in which multiple shorter IMRs occur within the span of a longer IMR. The longest IMRs coincide with protein functional motifs that are highly significant to the gene. Some mIMRs and rdIMRs overlap, and others are uniquely positioned in the gene.

Because there are so many IMRs, the question arises whether the coincidence of IMRs and functional motifs occurs by chance. This possibility is further complicated by the uncertainty of the boundaries of functional motifs, which becomes apparent in the detailed annotation in the Additional File 1.

Functional motifs have been determined primarily through the study of engineered mutants. However, a slightly different experimental design seems to have frequently led to the identifcation of a slightly different functional motif. Additionally, there is the possibility that a motif may not be complete. Therefore it is unlikely that a probability for the coincidence of IMRs with functional motifs can be computed. However, when IMRs are identified, solely on the basis of length, the longest of them coincide with key functional motifs in the protein. The relationship between length and significance first becomes apparent in the polyprotein, but persists independently in each of the mature proteins.

It is less problematic to identify the position of protein structural elements, although, again, differences in experimental design may result in slightly different boundaries for helices, turns and β-sheets (see Additional File 1). In this study, the ends of rdIMRs were found to coincide with the ends of protein structural elements over a range of about three nucleotides, a result consistent with a previous study of monomeric proteins. In HIV-1 Gag, this property is also found in mIMRs, and reverse dinucleotide pairs terminate 55% of the longest mIMRs in Gag. This feature may be related to the structural nature of Gag proteins, a premise that would also be consistent with the absence of mIMRs in highly mobile segments of MA and CA.

IMRs at low levels of symmetry begin and/or end at cleavage positions in the protein. IMRs having higher levels of symmetry coincide with PSEs and significant functional motifs in the protein. The highest levels of symmetry delineate essential functional sites in the protein. Analysis of the distribution of IMRs in the Gag polyprotein indicates that the gene sequence exhibits a high degree of regularity, is stabilized by multiple levels of mirror symmetry, and consists of sequence segments that are specifically associated with functional attributes of the protein segments that they translate.

## Conclusion

Key structural and functional features of each protein are almost always translations of IMRs. The distribution, by length, of the segments that translate the most significant motifs in each protein over the span of the polypeptide indicates that the polypeptide is the functional unit of organization for DNA motifs. The five longest mIMRs in *gag *that are ≥ 50% symmetric each translate the most significant protein motif in a different cleavage product.

Various thresholds for DNA symmetry differentiate functional and structural properties of the polyprotein that is translated. MIMRs that are ≥33% symmetric start or stop at cleavage positions, and positions that are functionally related in the mature proteins. IMRs that are ≥50% symmetric coincide with most of the functional motifs in the mature proteins. At ≥ 66% symmetry, the distribution of mIMRs and rdIMRs overlap and most of these motifs are related to structural features.

The frequency and distribution of IMRs in HIV-1 Gag indicates that DNA symmetry is a fundamental property of protein coding DNA and that different levels of symmetry are associated with different functional aspects of gene and protein. The interaction between DNA and protein structure and function is precise and interwoven over the entire length of the protein. The distribution of mIMRs and rdIMRs and their relationship to structural and functional motifs in the protein that they translate, suggest that DNA-driven processes, including selection for mirror repeats, may be a constraining factor in molecular evolution.

## Methods

### Sequence analysis

The HIV-1 gag sequence used for this analysis is HXB2_LAI_IIIB_BRU [GenBank:K03455], the most commonly used reference sequence for the HIV-1 genome [2]. All numbering in this paper refer to positions from the start of *gag*, unless stated otherwise.

### Determination of mIMRs and rdIMRs

The mIMRs and rdIMRs were determined for the differential cleavage products of HXB2 Gag: the polyprotein, the segments at the first cleavage – MA-CA-p2 and NC-p1-p6 – and MA, CA, NC, p6 and spacer proteins p2 and p1. MIMRs were evaluated at symmetry criteria of ≥ 33%, 45%, 50%, 55% and 66%; rdIMRs were evaluated at ≥50% and ≥66%.

### Evaluation of the coincidence of IMRs with PSEs

The coincidence of rdIMRs with PSEs was evaluated for the entire polyprotein and separately for each of its cleavage products. Because a high number of sub-strings might contribute to a false positive for the correlation of the ends of PSEs and IMRs, the number of IMRs was reduced by sequentially eliminating shorter lengths of IMRs, and testing whether the Fisher's exact test (FET) remained significant. The length of IMRs that have a positive FET correlation when all shorter IMRs are removed is identified as the "essential value"; this value was determined for each cleavage product.

The p6 region was not included in the rdIMR-PSE analysis because its tertiary structure has not been determined.

### Detailed annotation of Gag combined IMRs and functional and structural data

The sequence motifs of experimentally determined functional and structural data, and the sequence positions of the translations of mIMRs and rdIMRs were summarized and compared. Observed and expected frequencies of mIMRs and rdIMRs were determined. The largest IMRs were mapped to 3D structures from the NCBI Structure Database [19].

## Abbreviations

**IMR: **imperfect mirror repeat

**mIMR: **maximal imperfect mirror repeat

**rdIMR: **reverse dinucleotide imperfect mirror repeat

**MA: **matrix

**CA: **capsid

**SP1: **p2

**NC: **nucleocapsid

**PSE: **protein structural element

**L1: **refers to largest IMR for a particular sequence span

**FET: **Fisher's exact test

**PDB: **Protein Data Bank

## Competing interests

The author(s) declare that they have no competing interests.

## Authors' contributions

DML performed all computer-based analysis. DML wrote the manuscript and approved its final copy.

## Supplementary Material

Additional file 1 **Functional, structural and IMR motifs in Gag (HXB2)**. This table compares experimentally determined structural and functional positions of the Gag sequence with IMRs. The Gag sequence has a grey background. Annotations based on experimental evidence occur above the sequence; those that are translated by IMRs are bolded. The secondary structure of the sequence (its PDB file indicated to the right) is below the sequence (H = helix, B = residue in isolated beta bridge, E = extended beta strand, G = 310 helix, T = hydrogen bonded turn, S = bend). Below the structural information are the protein translations of DNA-IMRs identified in this study; to the right are this author's interpretation of the relationship between the indicated IMR and the known function indicated above the sequence. The IMR number indicates its rank, according to length. A hatch mark (#) indicates an mIMR; a dollar sign ($) indicates an rdIMR. Sequences that are protein translations of mIMRs are in bold letters. In order to simply the descriptions of function or structure for each motif, the earliest publication is referenced; if subsequent findings for the motif substantially altered interpretation, the motif is repeated with the new reference. References for this file are available in additional file 2. Click here for file

Additional file 2 **References for Additional file **1, **not listed in main manuscript**. References cited solely in **Additional file **1 are listed in this document. Click here for file
