# Supplementary material for: Imperfect DNA mirror repeats in the gag gene of HIV-1 (HXB2) identify key functional domains and coincide with protein structural elements in each of the mature proteins
Source: Virol J. 2007 Oct 26;4:113. doi: 10.1186/1743-422X-4-113 (PMC2211468; doi:10.1186/1743-422X-4-113)
Supplement: Additional file 1 — Functional, structural and IMR motifs in Gag (HXB2). This table compares experimentally determined structural and functional positions of the Gag sequence with IMRs. The Gag sequence has a grey background. Annotations based on experimental evidence occur above the sequence; those that are translated by IMRs are bolded. The secondary structure of the sequence (its PDB file indicated to the right) is below the sequence (H = helix, B = residue in isolated beta bridge, E = extended beta strand, G = 310 helix, T = hydrogen bonded turn, S = bend). Below the structural information are the protein translations of DNA-IMRs identified in this study; to the right are this author's interpretation of the relationship between the indicated IMR and the known function indicated above the sequence. The IMR number indicates its rank, according to length. A hatch mark (#) indicates an mIMR; a dollar sign ($) indicates an rdIMR. Sequences that are protein translations of mIMRs are in bold letters. In order to simply the descriptions of function or structure for each motif, the earliest publication is referenced; if subsequent findings for the motif substantially altered interpretation, the motif is repeated with the new reference. References for this file are available in additional file 2. [file 1743-422X-4-113-S1.pdf]

SEQUENCE	PROTEIN	IMR	FUNCTION OR STRUCTURE
LERFA**VNPGL**__	L41..V143		forms tether between MA at viral membrane and capsid [24]
V	V34		V34I reverses Env mutation which eliminated infectivity [25]
V	V34		interacts with gp-41 H2 to promote Env incorporation [25]
LKHIVW**ASRE LER**	L31..R43		acts together with basic residues to bind membrane surface [13]
L**KHIVWAS**RE LER	L31..R43		MA-H2 helix [8]
G	G25		silent mutation a75g reduces virion production [26]
GKKKYK LKH	G25..H33		nuclear localization signal 1 (NLS1) [11]
**PGGKKKYK LKH**	P23..H33		basic residues target and bind Gag to plasma membrane [9]
RPGGKKKYK	R22..K30		T22..K30 and I92..V95 affect structural orientation of MA-H5 [15]
IR L KQY	I19..G71		3-strand mixed b-sheet essential to membrane binding [27]
KIR LRPGGKKKYK LKH	K18..K31		mutations retarget particle formation to Golgi or post-Golgi vesicles [28]
**WEKIR L**RPGGKKKYK LKHIVWASRE LERFAVNPGL__	W16..E99		deletion redirects assembly and budding to endoplasmic reticulum [29]
R K R R KKK K KH R R	R15..R43		basic residues target and bind Gag to plasma membrane [9]
R K R R KKK K KH R R	R15..R43		basic region of MA is required for binding to EF1α [30]
RWEKIR LRPGGKKKYK LKHIVWASRE LER	R15..R43		substitution of > 3 non-basic for basic AA block virus production [9]
GELDRWEKIR LRPGG LKHIVWASRE LERFAV	G11..V46		may modulate myristoylation effects [10]
GELDRWEKIR LRPGG LKHIVWASRE LERFAV	G11..V46		deletions in this segment eliminate incorporation gp160 into virion [10]
GELDRWEKIR LRPGG LKHIVWASRE LERFAV	G11..V46		Calmodulin-binding domains [10]
G GELDRW	G10..W16		N-terminal a-helix of MA is binding site for AP-3 δ subunit [31]
G GELDRW	G10..W16		required for Gag to reach the MBV compartment [31]
G GELDRW	G10..W16		H1-MA helix [8]
V L	V6, L49		V6R, L49D mutations reduce infectivity [32]
VL L __	V7..K98		loss-of-function mutations V7R, L8AI restored by L21K and K98E [33]
S	S6I		S6I mutation results in no detectible virus [34]
GARAS	G2..S6		conformationally labile structure [8]
G	G2		myristic acid linked to G2 is essential to virus assembly [35]
G	G2		inhibitors of fatty acid synthesis prevent myristoylation [35]
MGARASV**LSG GEL**DRWEKIR LRPGGKKKYK LKHIVWASRE LERFAVNPGL	M1..L50		EF1α binding site [30]
MGAR**ASVLSG G**ELDRWEKIR LRPGGKKKYK LK	M1..K32		strong membrane-targeting signal - basic domain [36]
**MGARASV**	M1..V7		minimum signal required for myristoylation [37]
/ p17 matrix start			
1-MGARASVLSG GELDRWEKIR LRPGGKKKYK LKHIVWASRE LERFAVNPGL	M1..Y132		matrix protein
SS H HHHHHHSSSB STTT B HHHHHHHHHH HHHTTS GGG	1L6N		[8]
HH HHHHHHH HHHHHHHHH HHH HHHH	2GOL		[38]
H1-MA H2-MA			
NPGLLE	N47..E52	$9-MA	?
VNPGLLE	V46..S52	$2-MA	tether between MA at viral membrane and capsid
ASRE LER	A37..R43	$4-MA	binds HIV to plasma membrane; part of calmodulin-binding region
KHIVWAS	K32..S38	$8-MA	binds HIV to plasma membrane; part of calmodulin-binding region
GKKKYK LKHIVW	G25..W36	$3-gag	nuclear localization signal 1 (NLS1)
PGGKKKYK LKH	P23..H33	$7-gag	basic residues target and bind Gag to plasma membrane
WEKIR L	W16..L21	$6-MA	mutations retarget or block particle formation
LSG GEL	L8..L13	$11-MA	?
ASVLSG G	A5..G11	$27-gag	links myristoylation and calmodulin-binding region
MGARASV	M1..V7	$1-MA	minimum signal required for myristoylation

Additional File 1.

SEQUENCE	PROTEIN	IMR	FUNCTION OR STRUCTURE
__ K	V7..K98		loss-of-function mutations V7R, L8AI restored by L21K and K98E [33]
TKEA__	T97..A120		MA-H5 projects into center of virion; related to viral entry [13]
**TKEA__**	T97..A120		MA-H5 helix [8]
IEIV	I92..V95		T22..K30 and I92..V95 affect structural orientation of MA-H5 [15]
**RIEIKD**	R91..D96		connecting loop with highly variable charge (+ x - x + -)
C	C68		C68S mutation reduces infectivity [32]
LYCVH	L85..H89		L85R, Y86G, C87D, V88E, H89G eliminate virus replication [34]
**LYCVH**	L85..H89		mutations retarget assembly to trans- or post-Golgi vesicles [39]
EE**LRSLYN TVATLYC**VHQ	E73..Q90		central, buried helix contacts all other secondary elements [13]
**EELR**SLYN TVATLYCVHQ	E73..Q90		MA-H4 helix [8]
SLQT G	S67..G71		3-strand mixed b-sheet essential to membrane binding [27]
PSLQT G	P66..G71		each end a hinge to facilitate structural transformation at maturation [15]
**PSLQT G**	P66..G71		310-helix undergoes conformational change upon trimerization [8]
GC I D A			G56E, C57D, C57S, I60E, D96L, A99E eliminate replication [34]
SEG**CRQI LGQLQPS**L	S54..L68		mutations in this helix prevent MA trimerization, virus assembly [40]
SE**GCRQI LG**QLQPSL	S54..L68		MA-H3 helix [8]
LL C YC	L50..C87		L50A-L51A, C57S, Y86R-C87S prevent particle formation [41]
__LETSEGCRQI LGQLQPSLQT GSEELRSLYN TVATLYCVHQ RIEIKDTKE__	L41..V143		forms tether between MA at viral membrane and capsid [24]
__**LETSEGCR**QI LGQLQPSLQT GSEELRSLYN TVATLYCVHQ RIEIKDTKE	W16..E99		deletion redirects assembly and budding to endoplasmic reticulum [29]
51-LETSEGCRQI LGQLQPSLQT GSEELRSLYN TVATLYCVHQ RIEIKDTKEA	M1..Y132		matrix protein
SSSHHHHHHH HHHHHHHHSS S HHHHHHHH HHHHHHHHHH T SBHHHH	1L6N		[8]
GGGGG G	310 helix		[15]
H HHHHHHHH HHHHHHHHH HHHHHHHHH HHHHHHHHHH H HHHHH	2GOL		[38]
H3-MA H4-MA H5-MA_	1L6N		[8]
**RIEIKDTKEA__**	**R91..T122**	#1-gag	turn holds MA-H5 in position required for MA-CA cleavage
Q RIEIKDTKEA	Q90..A100	$5-gag	
**Q RIEIKDTKEA__**	**H89..N109**	#18-gag	~ #1-gag m-IMR
**HQ RIEIKDTKEA__**	**H89..Q116**	#4-gag	~ #1-gag m-IMR
ATLYCVHQ RI	A83..I92	$11-gag	
LRSLYN TVATLYC	L78..C87	$12-gag	MA-H4 is central to 3D structure and contacts all other secondary elements
PSLQT GSEELR	P66..R76	$10-gag	essential to structural transformation at maturation
GQLQPSLQT G	G62..G71	$9-gag	COOH end of MA-H3 helix
CRQI LGQLSPS	C57..S67	$6-gag	MA-H3 helix, C57S prevents particle formation
GCRQI LG	G56..G62	$7-MA	G56E, C57D, C57S, I60E eliminate replication
LETSEGC	L51..C57	$3-MA	L50A-L51A, C57S, Y86R-C87S prevent particle formation

SEQUENCE	PROTEIN	IMR	FUNCTION OR STRUCTURE
PR__	P149..K162		CA-H1-H2 form interface 2, a structurural component of viral core [14]
PR__	P149..K162		CA-H1 helix [8]
PIVQNIQG QMVHQ	P133..Q145		b-hairpin. Folds the charged P1 back into CA protein [16]
PIVQNIQG QMVHQ	P133..Q145		folded, charged P1 forms buried salt bridge with Asp51 [16]
PIVQNIQG QMVHQAISPR__	P133..D183		refolded CA-CA interface essential conical core assembly [16]
PIVQNIQG QMVHQAISPR__	P133..278		mediates hexamer formation in viral capsid [38]
PIVQNIQG QMV	P133..V143		conformation differs substantially in immature vs mature capsid [8]
PIVQN QMVHQ	P133..Q145		P133..N137, Q141..Q145 form antiparallel b-hairpin packs abainst H6 [8]
P	P133		disruption abolishes infectivity in Moloney murine leukemia virus [16]
P	P133		forms salt bridge with D183 [42]
YPIV	Y132..V135		potential tyrosine-based sorting signal [43]
**GHSNQVSQ NYPIVQN**IQG QMV	G123..V143		conformationally labile structure [8]
**KKKAQ**	K112..Q116		potential phosphorylation site [43]
S	S111		highly conserved protein kinase C (PKE) phosphorylation site [44]
	S111		may function in reversible control of latent provirus activation [44]
K SKKK	K110..K114		nuclear localization signal 2 (NLS2) [11]
K SKKK	K110..K114		interacts with cellular NLS receptor, karyopherin alpha [45]
**K SKKK**	K110..K114		nuclear localization signal essential to PIC entering nucleus [46]
**EEEQNK SKKKAQQAAA DTG**HSNQVSQ NY	E105..Y132		distinct function from rest of MA; deleterious mutations block viral entry [13
__LDKIEEEQNK SKKKAQQAAA	T97..A120		MA-H5 projects into center of virion; related to viral entry [13]
**__LDKIEEEQNK SKKKAQQAAA**	T97..A120		MA-H5 helix [8]
__LDKIEEEQNK SKKKAQQAAA DTGHSNQVSQ NYPIVQNIQG QMV	L41..V143		tether between MA at viral membrane and interior capsid [24]
\/			2cd step in maturation [3]
end p17 matrix \/ start p24 capsid			CA separated from membrane by MA-CA cleavage [3]
101-LDKIEEEQNK SKKKAQQAAA DTGHSNQVSQ NY	M1..Y132		matrix protein
________PIVQNIQG QMVHQAISPR	P133..L363		capsid protein
HHHHHHHHHH HHHHHHHHHH T SS S HH	1L6N		[8]
EEE SSS E EE HH	1GWP		[8]
HHHHH EEEE EEEEE HHH	2GOL		[38]
__H5-MA H1-CA	P149..K162		CA-H1 helix [8]
SQ NYPIVQN	S129..N137	$18-gag	cleavage site MA-CA
GHSNQV	G123..V128	$12-MA	start of conformationally labile structure near cleavage site
K SKKKAQ	K110..Q116	$5-MA	nuclear localization signal
EQNK SKK	E107..K113	$10-MA	MA-H5 helix, nuclear localization signal
**ALDKIEEEQNK SKKKAQQAAA DTGH**	**A100..H124**	#12-gag	MA-H5 helix
**____LDKIEEEQNK SKKKAQQAAA DT**	**91R..T122**	#1-gag	MA-H5 helix
**____LDKIEEEQNK SKKKAQ**	**H89..Q116**	#4-gag	MA-H5 helix
**____LDKIEEEQN**	**H89..N109**	#18-gag	~ #1-gag m-IMR

SEQUENCE	PROTEIN	IMR	FUNCTION OR STRUCTURE
QAAMQM__	A197..V215		CA-H4 helix [8]
D	D183		forms salt bridge with P133 in mature CA [42]
**PQDLNTMLNT** **QAAMQM__**	P181..V215		CA-H3-H4 pack lengthwise with CA-H1-H2-H7 to form viral core [14]
PQDLNTMLNT	P181..T190		CA-H3 helix [8]
M	M39D		completely abolished capsid cylinder formation in vitro [16]
PEVIP MFSAL	P166..L175		COOH-terminal of H2 helix packs against MHR [47]
PEVIP MFSAL	P166..L175		CA-H2 helix [8]
__TLNAWVKVVE EK **PEVIP MF**SAL	P149..K162		CA-H1-H2 form interface 2, a structurural component of viral core [14]
__TLNAWVKVVE EK	P149..K162		CA-H1 helix [8]
__TLNAWVKVVE EKA**FSPEVIP MF**SALSEGAT PQD	P133..D183		refolds to create a CA-CA interface essential to assemble core [16]
__TLNAWVKVVE EKAFSPEVIP MFSALSEGAT PQDLNTMLNT VGGHQAAMQM__	P133..S278		mediates hexamer formation in viral capsid [38]
151-TLNAWVKVVE EKAFSPEVIP MFSALSEGAT PQDLNTMLNT VGGHQAAMQM	P133..L363		capsid protein
HHHHHHHHHH HHSSSHHHHT HHHHHTTT HHHHHHHHHH S HHHHHH	1L6N		[8]
HHHHHHHHHH HHH HHH HHHHHH H HHHHHHHHHH HHHHHHH	2GOL		[38]
H1-CA H2-CA H3-CA H4-CA__			
AMQM__	A197..T204	$24-gag	NH3 end of CA-H4 helix
NT VGGHQ	N189..Q195	$2-CA	connects CA-H3 and CA-H4 helices
**PQDLNTMLNT VGGHQAAMQMLK**	**P181..K202**	#15-gag	CA H3 and H4 helices; constitutent of viral core
FSPEVIP MF	F164..F172	$16-gag	folds agains MHR

SEQUENCE	PROTEIN	IMR	FUNCTION OR STRUCTURE
LQEQIGWM	L243..M250		packs against P133..N137, Q141..Q145 in mature CA [8]
**LQE**QIGWM	L243..M250		CA-H6 helix [8]
GSDIA	G233..A237		CA-H5 helix [8]
RGSDIAG	R232..R238		abrogates antigen-induced responses in cultures of human PBMC [48]
Q	Q227		CA Q95, component of CypA interaction [49].
GP	G221..P222		essential for CyPA-binding [50]
HA GPIA	H219..A224		interacts with (human) CypA which is essential for HIV infectivity [51]
**PVHA GPIAPGQMRE PR**GSDIA**GTT S**	P217..S241		exposed loop thought be be located on surface of virion core [12]
PVHA GPIAPGQMRE P	P217..P231		conformationally flexible loop [8]
IN**EEAA EWDRVHPVHA GPI**APGQMRE PRGSDIAGTT S	I205..S241		cyclophilin A(CypA) binding site; CypA may promote CA uncoating [12]
__**LKET**INEEAA EWDRV	P181..V215		CA-H3-H4 pack lengthwise with CA-H1-H2-H7 to form viral core [14]
__LKETINEEAA EWDRV	Q195..V215		CA-H4 helix [8]
__LKETINEEAA EWDRVHPVHA GPIAPGQMRE PRGSDIAGTT STLQEQIGWM__	P133..S278		mediates hexamer formation in viral capsid [38]
201-LKETINEEAA EWDRVHPVHA GPIAPGQMRE PRGSDIAGTT STLQEQIGWM	P133..L363		capsid protein
HHHHHHHHHH HHHHHS S SS HHHHHTSS S HHHHHHHH	1L6N		[8]
HHHHHHHHHH HHHHH HHHHHH	2GOL		[38]
_H4-CA H5-CA H6-CA			
GWM__	**G248..M276**	#2-gag	CA-H6 through CA-H7 helix
GTT STLQE	G238..E245	$28-gag	links CA-H5 and -H6 helices
GQMRE PR	G226..R232	$4-CA	CypA interaction; part of loop on surface of virion core
PVHA GPIAP	P217..P225	$22-gag	CypA interaction; part of loop on surface of virion core
HPVHA GPI	H216..I223	$3-CA	CypA interaction; part of loop on surface of virion core
EEAA EWDRV	E207..V215	$14-gag	CypA interaction
____LKET	A197..T204	$24-gag	N end of CA H4 helix; constituent of viral core

Additional File 1.

SEQUENCE	PROTEIN	IMR	FUNCTION OR STRUCTURE
EPFRDYVDRF__	E291..A306		CA-H8 helix [8]
K EPF	K290..F293		deletion causes major defect in particle formation [52]
PK EPFR			motif recognized by class II SH3 domains [43]
RQGPK EP	R286..P292		motif recognized by class I SH3 domains or noncanonical specificity [43]
IRQG	I282..G285		deletion causes major defect in particle formation [52]
DIRQGPK EPFRDYVDRFYKT	D284..T303		major homology region (MHR) [53]
DIRQGPK EPFRDYVDRFYKT	D284..T303		MHR is essential for particle formation [54]
DIRQGPK EPFRDYVDRFYKT	D284..T303		packs against the COOH-terminal of H2 helix [47]
Q	Q287		invariant residue; mutation blocks viral assembly [55]
T SIL			FHA domain interaction motif
PT SILDIRQGPK EPFRDYVDRF__	P279..N432		minimal internalization sequence of Gag [56]
PT SIL	P279..L283		conformationally labile structure [8]
PT SILDIRQGPK EPFRDYVDRF	P279..L363		mediates association adjacent CA hexamers in core [38]
**SPT SILDIRQGPK EPFRDY**VDRF__	S278..L363		required for capsid dimerization and viral assembly [47]
SPT SIL	S278..L283		necessary for formation of high-affinity capsid dimer interface [47]
SP	S278..P279		disordered residues that link the N- and C-terminal CA domains [57]
VGE IYKRWIILGL NKIVRM	V258..M276		CA-H7 stabilizes interface 1 (planar strips) of viral core [14]
**VGE IYKRWIILGL NKIVRM**	V258..M276		CA-H7 helix [8]
N			CA-N253. Essential component of CypA interaction [49]
TNNPPIPVGE IYKRWIILGL NKIVRMYS	P133..S278		mediates hexamer formation in viral capsid [38]
251-TNNPPIPVGE IYKRWIILGL NKIVRMYSPT SILDIRQGPK EPFRDYVDRF	P133..L363		capsid protein
SSSS HHH HHHHHHHHHH HHHHHHSSTT	1L6N		[8]
HHHH HHHHHHHHHH HHHHHHHH	2GOL		[38]
GGGG SS HHHHHHHHHH	1BAJ		[57]
H7-CA H8-CA			
RF_	R299..A306	$19-gag	end MHR
SPT SILD	S278..D284	$7-CA	necessary for formation of dimer interface
**NKIVRMYSPT SILDIRQGPK EPFRDY**	**N271..Y296**	#10-gag	potential phosphorylation and major homology region
**NKIVRMYSPT SILDIRQGPK E**	**N271..E291**	#16-gag	~ #10-gag
IPVGE IYKRWIIL	I256..L268	$2-gag	CA-H7 helix
**PPIPVGE IYKRWIILGL NKIVRMYSPT**	**P255..T280**	#8-gag	~ #2-gag
**NPPIPVGE IYKRWIILGL NKIVRMYSPT**	**N253..T280**	#5-gag	~ #2-gag
**__TNNPPIPVGE IYKRWIILGL NKIVRM**	**G248..M276**	#2-gag	CA-H6 helix

Additional File 1.

SEQUENCE	PROTEIN	IMR	FUNCTION OR STRUCTURE
ACQ	A349..Q351		deletion causes major defect in particle formation [52]
**LEEMMTAC**	L343..C350		CA-H11 helix [8]
LGP	L337..P340		deletion causes major defect in particle formation [52]
DC KTIL	D329..L333		endocytosis signal [56]
P**DC KTILKAL**	P328..L337		CA-H10 helix [8]
**QNAN**	Q324..N327		deletion causes major defect in particle formation [52]
TET LL	T318..L322		endocytosis signal [56]
QEVKN**WMTET LLV**Q	Q311..Q324		CA-H9 helix [8]
QA**S QEVKNW**MTET LLVQNANPDC KTILKALGPA ATLEEMMTAC__	Q308..Q351		interacts with LysRS, leads to incorporation of LysRS into viron[58]
EQA	E307..A309		deletion causes major defect in particle formation [52]
__YKTLRA**EQAS QEVKNWMTET LLVQNANPDC KT**ILKALGPA ATLEEMMTAC__	P279..N432		minimal internalization sequence of Gag [56]
__YKTLRAEQAS QEVKNWMTET LLVQNANPDC KTILKALGPA ATLEEMMTAC__	P279..N432		promotes multimerization of Gag [56]
__YKTLRAEQAS QEVKNWMTET LLVQNANPDC KTILKALGPA ATLEEMMTAC__	S278..L363		required for capsid dimerization and viral assembly [47]
__YKTLRAEQAS QEVKNWMTET LLVQNANPDC KTILKALGPA ATLEEMMTAC__	P279..L363		mediates association adjacent CA hexamers in core [38]
301-YKTLRAEQAS QEVKNWMTET LLVQNANPDC KTILKALGPA ATLEEMMTAC	P133..L363		capsid protein
HHHHHHTT HHHHHHHHHT HHHHTS HHH HHHHHHH SS HHHHHHH	1BAJ		[57]
__H8-CA H9-CA H10-CA H11-CA			
TACQ__	T348..P356	$23-gag	interacts with LysRS
MTACQ__	M347..V353	$06-CA	interacts with LysRS
LEEMMTACQ	L343..Q351	$21-gag	interacts with LysRS
**DC KTILKALGPA ATLEEMMTACQG**	**D329..G352**	#14-gag	endocytosis signal, CA H11 helix
LVQNANPDC	L322..C330	$20-gag	deletion causes major defect in particle formation [52]
WMTET LLV	W316..V323	$05-CA	1st endocytosis signal, interacts with LysRS
QEVKNWMT	Q311..T318	$25-gag	interaction with LysRS
S QEVKNW	S310..W316	$09-CA	interacts with LysRS, leads to incorporation of LysRS into viron
**EQAS QEVKNWMTET LLVQNANPDC KT**	**E307..T332**	#11-gag	endocytosis signal, CA H9 helix, interaction with LysRS
LRAEQAS	L304..S310	$08-CA	spans CA-H8-H9 helices
RFYKTLRA	R299..A306	$19-gag	end MHR

SEQUENCE	PROTEIN	IMR	FUNCTION OR STRUCTURE
CFNCGKEGHC	C392..C401		1st cys-his box (Cen et al, 1999); binds one Zn ion [10]
R..RK	R380..K383		mutation inhibits tRNA-lys3 annealing, reverse transcription [59]
R R	R380A, R384A		critical residues for Gag-Gag interaction [60]
K K	K383, K386		essential for efficient RNA packaging [61]
R R RK K K __	R380..R429		basic residues essential to ABCE1 binding, Gag multimerization [62]
R	R380		essential for viral replication [61]
MQR GNFR	M378..R384		I-domain [63-64]
MQR GNFRNQRK	M378..K388		lack of I-domain results in monomeric Gag [60]
MQR GNFRNQRK	M378..K388		mediates Gag-Gag interaction, particle assembly [5]
MQR GNFRNQRK	M378..K388		interacts with human APOBEC3G [66]
MQR GNFRNQRKIV	Q379..V390		actin interaction domain in immature HIV [68]
MQR GNFRNQRKIV **KCFNCGKEGHC**_	M378..Q430		N-term basic region and Zn fingers essential aggregation ssDNA [69]
M**QR GNFRNQRKIV KCFNCGKEGH**__	M378..N432		EF1α binds to basic residues on NC [30]
TNSA	T371..A374		FHA domain interaction motif; glycosaminoglycan attachment site [43]
**AEAMSQV TNS**ATIM	A364..M377		p2 protein (SP1); budding is critically dependent on p2 [70]
**GHKA RVLAEAMSQV TNSATIMMQR G**	G357..G381		region increases affinity of NC for genomic RNA [71]
GHKA RVLAEAMSQV	G357..V370		spans p2; continuous α-helix between CA and NC[72]
**GVGGPG**	G354..G359		flexible C-term motif allows close association of p2 helices in dimer [70]
__QGVGGPGHKA RV	Q308..Q351		interacts with LysRS, leads to incorporation LysRS into viron[58]
__QGVGGPGHKA RVLAEAMSQV TNSATIMMQR GNFRNQRKIV KCFNCGKEGH__	P279..N432		minimal internalization sequence of Gag [56]
__QGVGGPGHKA RLV	S278..L363		required for capsid dimerization and viral addembly[47]
\/	L363..A364		3rd step in maturation, critical to formation normal cone-shaped core [3]
\/	L363..A364		Release of SP1 from CA is required for capsid condensation [3]
\/	L363..A364		SP1 may retain CA in immature conformation [3]
\/	M377..M378		1st step in maturation is cleavage at C terminus of SP1 [3].
\/	M377..M378		releasing NC leads to condensation of core [3]
__QGVGGPGHKA RVL	P279..L363		mediates association adjacent CA hexamers in core [38]
end p24 capsid \/ p2 or SP1 \/ start p7			
351-QGVGGPGHKA RVL	P133..L363		capsid protein
AEAMSQV TNSATIM	A364..M377		p2 protein (SP1)
MQR GNFRNQRKIV KCFNCGKEGH	M378..N432		p7 - nucleocapsid protein
____TTTSB TTHH HHHHHHHHHH HHHHHHHHHT TTTTTSSS	K359..Q379		1BAJ [57]
G GGGGGGG B TTT BSS	1F6U		[18]
**KCFNCGKEGH__**	**K391..G417**	#6-gag	Zn finger domain
**NQRKIV KCFNCGKEGH**	**N385..H400**	#2-NC	EF1α binding
V TNSATIMM	V370..M378	$17-gag	cleavage site p2-p7, phosphorylation
V TNSATIM	V370..M377	$1-CA	cleavage site p2-p7, phosphorylation
**MSQV TNSATIMMQR GNFRNQRKIV KC**	**M367..C392**	#9-gag	spans p2, I domain, increases affinity for genomic RNA, packaging, buddin
AEMSQV TNS	A364..S373	$13-gag	cleavage site, start of p2
KA RVLAEAM	K359..M367	$15-gag	cleavage site CA-p2
**PGHKA RVLAEAMSQV TNSATIMMQR GN**	**P356..N382**	#7-gag	spans p2, increases affinity for genomic RNA, packaging, budding
____QGVGGP	T348..P356	$23-gag	flexible C-terminal motif
____QGV	M347..V353	$6-CA	flexible C-terminal motif

Additional File 1.

SEQUENCE	PROTEIN	IMR	FUNCTION OR STRUCTURE
P P			P7 and P13 are essential for replication[73]
FL			slip site for GagPol
TER	T427..R429		PKC phosphorylation site essential to HIV packaging
__ CWKCGK**EG HCQMKDCTERQ**	M378..Q430		N-term basic region, Zn fingers essential to aggregation ssDNA [69]
**CWKCGKEG H**	C413..H421		2cd cys-his box (Cen et al, 1999); binds one Zn ion [10]
RK K	R409..K411		mutation inhibits tRNAlys3 primer annealing [59]
RK K	R409..K411		mutation inhibits initiationn reverse transcription [59]
K KGCWKCGKEG HQMK	K410..K424		selection AAA over AAG t-RNA-lys primers increases infectivity [74]
**RAPRK KG**	R406..G112		crucial to NC-RT binding, enhanced RT processivity [83]
I K	I24T, K26R		mutation reduces virus infectivity [66]
__ R R RK K K K K R	R380..R429		basic residues essential to ABCE1 binding, Gag multimerization [62]
__TARNC CWKCGKEG HQMKDCT	C413..T427		Zn finger motifs [43]
__TAR**NCRAPRK KGCWKCGKEG HQMKDCTERQ AN**	M378..N432		EF1α binds basic residues within this sequence [30]
__**TARNCRAPRK KGCWKCG**KEG HQMKDCTERQ AN	P279..N432		minimal internalization sequence of Gag [56]
L__	L450..Q500		p6 mediates virion budding from infected cells [76]
end p7 \/ p1 \/p6			
401-TARNCRAPRK KGCWKCGKEG HQMKDCTERQ AN	M378..N432		p7 - nucleocapsid protein
GGG S SEETTTTEET TTGGG S SS	1F6U		[18]
FLGKIWPS YKGRPGNFLQ	F433..F448		p1 protein (SP2); start is slip site
FREDLAFP QGKARERSSE	F433..F488		Gag-Pol TF
S YKGRPGNFL__	S440..A457	#	occurs as an L1 m-IMR only in the NC-SP2-p6 segment
S YKGRP	S440..P445	$2-p1	p1 protein (SP2)
KIWPS YK	K436..K442	$1-p1	start is slip site; p1 protein (SP2)
**GKIWPS YKGRPGNFL__**	**G435..E454**	#	occurs as an L1 m-IMR only in the NC-SP2-p6 segment
CTERQ ANF	C426..F433	$26-gag	cleavage site p7-p1
**EG HQMKDCTERQ ANFLGKIWPS YKGRPGN**	**E419..N447**	#3-gag	end of NC, SP2
CGKEG HQMKDCT	C416..T427	$4-gag	Zn finger motifs. 2cd cys-his box
RAPRK KGCWKCGKEG H	R406..H421	$1-gag	
**NCRAPRK KGCWKCGKEG HQMKDCTERQ AN**	**N404..M423**	#1-NC	EF1α binding
**TARNCRAPRK KGCWKCG**	**K391..G417**	#6-gag	EF1α binding

Additional File 1.

SEQUENCE	PROTEIN	IMR	FUNCTION OR STRUCTURE
**LR SL**F	L489..F493		LXXLF. important for Vpr packaging
LYPL L L	L482..L491		motif required for Alix-mediated budding [77]
K	K474		site of covalent SUMO-1 attachment [76]
TP	T471..P472		conserved site essential to virion detachment, infectivity [78]
T	T471A		incomplete separation from host cell membrane[79]
E ES	E460..S462		VPS37B, VPS28 bind to Tsg101; both required for HIV-1 release [80]
PTAPP	P455..P459		late (L) domain of Gag
PTAPP	P455..P459		required for completion of viral budding [81]
PTAPP	P455..P459		Tsg101 docking site, essential for budding [82-83]
PSAP	P455..P458		PSAP mutant potently inhibits HIV-1 particle release [84]
LSRPEPXAPPE ESFRFGXE	L449..E468		ubiquitin-gag conjugates found for this sequence [85]
LSRPEPTAPPE ESFRSGVETT **TPPQKQEPID KELYPLT**SLR SLFGNDPSSQ	L449..Q500		monoubiquitination regulates internalization endocytic pathway [86]
LSRPE**PTAPPE ESFRSGVETT TPPQK**QEPID KELYPLTSLR SLFGNDPSSQ	L449..Q500		p6 is monoubiquitinated [87]
__SRPEPTAPPE ESFRSGVETT TPPQKQEPID KELYPLTSLR SLFGNDPSSQ	L449..Q500		p6 mediates virion budding from infected cells [76]
end p6 \			
451-SRPEPTAPPE <u>ESFRSGVETT</u> <u>TPPQKQEPID</u> KELYPLTSLR SLF<u>GNDPSSQ</u>	L449..Q500		p6 protein; end of Gag
QTRANSPTRR ELQVWGRDNN SPSEAGADRQ GTVSFNF	F433..F488		Gag-Pol TF
PQV TLWQRPLVTI__	P489..		Pol protease
**LR SL**	L489..L492	rd-IMR	12 nt, 67% symmetry
T TPPQKQEPID KELYPLT	T470..T487	#	occurs as an L1 m-IMR only in the NC-SP2-p6 segment
E ESFRSGVETT	E460..T470	$8-gag	possible association with ubiquitin
**PTAPPE ESFRSGVETT TPPQK**	**P455..K475**	#17-gag	late (L) domain of Gag; separation from host cell membrane
__SRPEPTA	S440..A457	#	occurs as an L1 m-IMR only in the NC-SP2-p6 segment
__SRPE	G435..E454	#	occurs as an L1 m-IMR only in the NC-SP2-p6 segment
