# Supplementary material for: Imperfect DNA mirror repeats in the gag gene of HIV-1 (HXB2) identify key functional domains and coincide with protein structural elements in each of the mature proteins
Source: Virol J. 2007 Oct 26;4:113. doi: 10.1186/1743-422X-4-113 (PMC2211468; doi:10.1186/1743-422X-4-113)
Supplement: Additional file 2 — References for Additional file 1, not listed in main manuscript. References cited solely in Additional file 1 are listed in this document. [file 1743-422X-4-113-S2.doc]

**References for Manuscript and Additional file 1**

1. Lang DM: **Imperfect DNA mirror repeats in E. coli TnsA and other protein-coding DNA**. *Biosystems* 2005, **81(3):**183-207.

2. Korber BT, Foley BT, Kuiken CL, Pillai SK and Sodroski G: **Numbering Positions in HIV Relative to HXB2CG**. In *Human Retroviruses and AIDS 1998: A Compilation and Analysis of Nucleic Acid and Amino Acid Sequences*. Edited by Korber B, Kuiken CL, Foley B, Hahn B, McCutchan F, Mellors JW, and Sodroski J. Theoretical Biology and Biophysics Group, Los Alamos National Laboratory, Los Alamos, NM.

3. Wiegers K, Rutter G, Kottler H, Tessmer U, Hohenberg H, Krausslich HG: **Sequential steps in human immunodeficiency virus particle maturation revealed by alterations of individual Gag polyprotein cleavage sites.** *J Virol* 1998, **72(4):**2846-54.

4. Pettit SC, Moody MD, Wehbie RS, Kaplan AH, Nantermet PV, Klein CA, Swanstrom R. **Free in PMC The p2 domain of human immunodeficiency virus type 1 Gag regulates sequential proteolytic processing and is required to produce fully infectious virions.** *J Virol* 1994, **68(12):**8017-27.

5. Swanstrom RA and Wills JW: **Synthesis, assembly and processing of viral proteins**. In *Retroviruses* by Coffin JM, Hughes SH and Varmus HE. Cold Spring Harbor Laboratory Press, 1997:263-334.

6. Freed EO: **HIV-1 gag proteins: diverse functions in the virus life cycle.** *Virology* 1998, **251(1):**1-15.

7. Shehu-Xhilaga M, Kraeusslich HG, Pettit S, Swanstrom R, Lee JY, Marshall JA, Crowe SM, Mak J: **Proteolytic processing of the p2/nucleocapsid cleavage site is critical for human immunodeficiency virus type 1 RNA dimer maturation.**

*J Virol* 2001, **75(19):**9156-64.

8. Tang C, Ndassa Y, Summers MF: **Structure of the N-terminal 283-residue fragment of the immature HIV-1 Gag polyprotein.** *Nat Struct Biol* 2002, **9(7):**537-43.

9. Yuan X, Yu X, Lee TH, Essex M: **Mutations in the N-terminal region of human immunodeficiency virus type 1 matrix protein block intracellular transport of the Gag precursor.** *J Virol* 1993, **67(11):**6387-94.

10. Radding W, Williams JP, McKenna MA, Tummala R, Hunter E, Tytler EM, McDonald JM: **Calmodulin and HIV type 1: interactions with Gag and Gag products.** AIDS Res Hum Retroviruses 2000, **16(15)**:1519-25.

11. Bukrinsky MI, Haggerty S, Dempsey MP, Sharova N, Adzhubel A, Spitz L, Lewis P, Goldfarb D, Emerman M, Stevenson M: **A nuclear localization signal within HIV-1 matrix protein that governs infection of non-dividing cells**. *Nature* 1993, **365(6447):**666-9.

12. Luban J: **Absconding with the chaperone: essential cyclophilin-Gag interaction in HIV-1 virions**. *Cell 1996,* **87(7):**1157-1159.

13. Hill CP, Worthylake D, Bancroft DP, Christensen AM, Sundquist WI: **Crystal structures of the trimeric human immunodeficiency virus type 1 matrix protein: implications for membrane association and assembly.** *Proc Natl Acad Sci USA* 1996, **93(7):**3099-104.

14. Gamble TR, Vajdos FF, Yoo S, Worthylake DK, Houseweart M, Sundquist WI, Hill CP:**Crystal structure of human cyclophilin A bound to the amino-terminal domain of HIV-1 capsid.** *Cell* 1996, **87(7):**1285-94.

15. Massiah MA, Worthylake D, Christensen AM, Sundquist WI, Hill CP, Summers MF: **Comparison of the NMR and X-ray structures of the HIV-1 matrix protein: evidence for conformational changes during viral assembly.** *Protein Sc*i 1996, **5(12):**2391-8.

16. von Schwedler UK, Stemmler TL, Klishko VY, Li S, Albertine KH, Davis DR, Sundquist WI: **Proteolytic refolding of the HIV-1 capsid protein amino-terminus facilitates viral core assembly.** *EMBO J* 1998, **17(6):**1555-68.

17. Priel E, Aflalo E, Seri I, Henderson LE, Arthur LO, Aboud M, Segal S, Blair DG: **DNA binding properties of the zinc-bound and zinc-free HIV nucleocapsid protein: supercoiled DNA unwinding and DNA-protein cleavable complex formation.** *FEBS Lett* 1995, **362(1):**59-64.

18. Amarasinghe GK, De Guzman RN, Turner RB, Chancellor KJ, Wu ZR, Summers MF: **NMR structure of the HIV-1 nucleocapsid protein bound to stem-loop SL2 of the psi-RNA packaging signal. Implications for genome recognition.** *J Mol Biol* 2000, **301(2):**491—511.

19. Omichinski JG, Clore GM, Sakaguchi K, Appella E, Gronenborn AM: **Structural characterization of a 39-residue synthetic peptide containing the two zinc binding domains from the HIV-1 p7 nucleocapsid protein by CD and NMR spectroscopy.** *FEBS Lett* 1991, **292(1-2):**25-30.

20. Langsrud O: Fisher’s exact test [http://www.matforsk.no/ola/fisher.htm]

21. Kabsch W, Sander C: **Dictionary of protein secondary structure: pattern recognition of hydrogen-bonded and geometrical features.**

*Biopolymers* 1983, **22(12):**2577-637.

22. Zhou W, Parent LJ, Wills JW, Resh MD: **Identification of a membrane-binding domain within the amino-terminal region of human immunodeficiency virus type 1 Gag protein which interacts with acidic phospholipids.** *J Virol* 1994, **68(4):**2556-69.

23. **NCBI Structure Database** [http://www.ncbi.nlm.nih.gov/entrez/query.fcgi?CMD=search&DB=structure]

24. Wilk T, Gross I, Gowen BE, Rutten T, de Haas F, Welker R, Krausslich HG, Boulanger P, Fuller SD: **Organization of immature human immunodeficiency virus type 1.** *J Virol* 2001, **75(2):**759-71.

25. Murakami T, Freed EO: **Genetic evidence for an interaction between human immunodeficiency virus type 1 matrix and alpha-helix 2 of the gp41 cytoplasmic tail.** *J Virol* 2000, **74(8):**3548-54.

26. Hamano T, Matsuo K, Hibi Y, Victoriano AF, Takahashi N, Mabuchi Y, Soji T, Irie S, Sawanpanyalert P, Yanai H, Hara T, Yamazaki S, Yamamoto N, Okamoto T: **A single-nucleotide synonymous mutation in the gag gene controlling human immunodeficiency virus type 1 virion production.** *J Viro*l 2007, **81(3):**1528-33. Epub 2006 Nov 22.

27. Massiah MA, Starich MR, Paschall C, Summers MF, Christensen AM, Sundquist WI: **Three-dimensional structure of the human immunodeficiency virus type 1 matrix protein.** *J Mol Biol* 1994, **244(2):**198-223.

28. Ono A, Demirov D, Freed EO: **Relationship between human immunodeficiency virus type 1 Gag multimerization and membrane binding.** *J Viro*l 2000, **74(11):**5142-50.

29. Facke M, Janetzko A, Shoeman RL, Krausslich HG: **A large deletion in the matrix domain of the human immunodeficiency virus gag gene redirects virus particle assembly from the plasma membrane to the endoplasmic reticulum.** *J Virol* 1993, **67(8):**4972-80.

30. Cimarelli A and Luban J: **Translation elongation factor 1-alpha interacts specifically with the human immunodeficiency virus type 1 Gag polyprotein.** *J Virol* 1999, **73(7):**5388-5401.

31. Dong X, Li H, Derdowski A, Ding L, Burnett A, Chen X, Peters TR, Dermody TS, Woodruff E, Wang JJ, Spearman P: **AP-3 directs the intracellular trafficking of HIV-1 Gag and plays a key role in particle assembly.** *Cell* 2005, **120(5):**663-74.

32. Murakami T, Ablan S, Nagashima K, Komano J, Miyauchi K, Matsuda Z, Freed E, Yamamoto N: 2005. **Characterization of HIV-1 matrix mutants: effect on the early stage of infection.** In: Meeting, *Retroviruses*, Cold Spring Harbor, New York, May 24-29, 2005.

33. Saad JS, Loeliger E, Luncsford P, Liriano M, Tai J, Kim A, Miller J, Joshi A, Freed EO, Summers MF: **Point mutations in the HIV-1 matrix protein turn off the myristyl switch.** *J Mol Biol* 2007, **366(2):**574-585.

34. Freed EO, Orenstein JM, Buckler-White AJ, Martin MA: **Single amino acid changes in the human immunodeficiency virus type 1 matrix protein block virus particle production.** *J Virol* 1994, **68(8):**5311-20.

35. Bryant M and Ratner L:. **Myristoylation-dependent replication and assembly of human immunodeficiency virus 1.** *Proc Natl Acad Sci USA* 1990, **87(2):**523-7.

36. Zhou W, Parent LJ, Wills JW, Resh MD: **Identification of a membrane-binding domain within the amino-terminal region of human immunodeficiency virus type 1 Gag protein which interacts with acidic phospholipids.** *J Virol* 1994, **68(4):**2556-69.

37. Lee PP and Linial M: **Efficient particle formation can occur if the matrix domain of human immunodeficiency virus type 1 Gag is substituted by a myristylation signal.** *J Virol* 1994, **68(10):**6644-54.

38. Kelly BN, Howard BR, Wang H, Robinson H, Sundquist WI, Hill CP: **Implications for viral capsid assembly from crystal structures of HIV-1 Gag(1-278) and CA(N)(133-278).** *Biochemistry* 2006, **45(38):**11257-66.

39. Ono A, Orenstein JM, Freed EO: **Role of the Gag matrix domain in targeting human immunodeficiency virus type 1 assembly.** *J Virol* 2000, **74(6):**2855-66.

40. Morikawa Y, Zhang WH, Hockley DJ, Nermut MV, Jones IM: **Detection of a trimeric human immunodeficiency virus type 1 Gag intermediate is dependent on sequences in the matrix protein, p17.** *J Virol* 1998, **72(9):**7659-63.

41. Cannon PM, Matthews S, Clark N, Byles ED, Lourin O, Hockley DJ, Kingsman SM, Kingsman AJ: **Structure-function studies of the human immunodeficiency virus type 1 matrix protein, p17.** *J Virol* 1997, **71(5):**3474-83.

42. Gitti RK, Lee BM, Walker J, Summers MF, Yoo S, Sundquist WI: **Structure of the amino-terminal core domain of the HIV-1 capsid protein.** Science 1996, **273(5272):**231-5.

43. ELM. Eukaryotic Linear Motif Resource. http://elm.eu.org/about.html

44. Burnette B, Yu G, Felsted RL: **Phosphorylation of HIV-1 gag proteins by protein kinase C.** *J Biol Chem* 1993, **268(12):**8698-703.

45. Nadler SG, Tritschler D, Haffar OK, Blake J, Bruce AG, Cleaveland JS: **Differential expression and sequence-specific interaction of karyopherin alpha with nuclear localization sequences.** *J Biol Chem* 1997, **272(7):**4310-5.

46. Haffar OK, Popov S, Dubrovsky L, Agostini I, Tang H, Pushkarsky T, Nadler SG, Bukrinsky M: **Two nuclear localization signals in the HIV-1 matrix protein regulate nuclear import of the HIV-1 pre-integration complex.** *J Mol Biol* 2000, **299(2):**359-68.

47. Gamble TR, Yoo S, Vajdos FF, von Schwedler UK, Worthylake DK, Wang H, McCutcheon JP, Sundquist WI, Hill CP: **Structure of the carboxyl-terminal dimerization domain of the HIV-1 capsid protein.** *Science* 1997, **278(5339):**849-53.

48. Giacomini E, Chersi A, Giordani L, Luzzati A: **Possible role of the plasminogen receptor as a site of interaction of the human immunodeficiency virus p24 immunosuppressive heptapeptide Ch7 with the host immune system.** *Scand J Immunol* 2000, **51(2):**164-7.

49. Agarwal PK: **Cis/trans isomerization in HIV-1 capsid protein catalyzed by cyclophilin A: insights from computational and theoretical studies.** Proteins 2004, **56(3):**449-63.

50. Braaten D, Ansari H, Luban J: **The hydrophobic pocket of cyclophilin is the binding site for the human immunodeficiency virus type 1 Gag polyprotein.** *J Virol* 1997, **71(3):** 2107-13.

51. Sherry B, Zybarth G, Alfano M, Dubrovsky L, Mitchell R, Rich D, Ulrich P, Bucala R, Cerami A, Bukrinsky M: **Role of cyclophilin A in the uptake of HIV-1 by macrophages and T lymphocytes.** *Proc Natl Acad Sci U S A* 1998, **95(4):**1758-63.

52. Dorfman T, Bukovsky A, Ohagen A, Hoglund S, Gottlinger HG: **Functional domains of the capsid protein of human immunodeficiency virus type 1.** *J Virol* 1994, **68(12):**8180-7.

53. Wills JW and Craven RC: **Form, function, and use of retroviral Gag proteins.** *AIDS* 1991, **5(6):**639-654.

54. Mammano F, Ohagen A, Hoglund S, Gottlinger HG: **Role of the major homology region of human immunodeficiency virus type 1 in virion morphogenesis.** J Virol 1994, **68(8):**4927-36.

55. Ebbets-Reed D, Scarlata S, Carter CA: **The major homology region of the HIV-1 gag precursor influences membrane affinity.** *Biochemistry* 1996, **35(45):**14268-75.

56. Lindwasser OW, Resh MD: **Human immunodeficiency virus type 1 Gag contains a dileucine-like motif that regulates association with multivesicular bodies.** *J Virol* 2004, **78(11):**6013-23.

57. Worthylake DK, Wang H, Yoo S, Sundquist WI, Hill CP: **Structures of the HIV-1 capsid protein dimerization domain at 2.6 A resolution.** *Acta Crystallogr D Biol Crystallogr* 1999, **55(Pt 1):**85-92.

58. Javanbakht H, Halwani R, Cen S, Saadatmand J, Musier-Forsyth K, Gottlinger HG and Kleiman L: **The interaction between HIV-1 Gag and human lysyl-tRNA synthetase during viral assembly.** *J Biol Chem* 2003, **278(30):**27644-27651.

59. Huang Y, Khorchid A, Gabor J, Wang J, Li X, Darlix JL, Wainberg MA, Kleiman L: **The role of nucleocapsid and U5 stem/A-rich loop sequences in tRNA(3lys) genomic placement and initiation of reverse transcription in human immunodeficiency virus type 1**. *J Virol* 1998, **72(5):3**907-3915.

60. Derdowski A, Ding L, Spearman P: **A novel fluorescence resonance energy transfer assay demonstrates that the human immunodeficiency virus type 1 Pr55Gag I domain mediates Gag-Gag interactions.** *J Virol* 2004, **78(3)**:1230-42.

61. Poon DT, Wu J, Aldovini A: **Charged amino acid residues of human immunodeficiency virus type 1 nucleocapsid p7 protein involved in RNA packaging and infectivity.** *J Virol* 1996, **70(10):**6607-16.

62. Lingappa JR, Dooher JE, Newman MA, Kiser PK, Klein KC: **Basic residues in the nucleocapsid domain of Gag are repuired for interaction of HIV-1 Gag with ABCE1 (HP68), a cellular protein important for HIV-1 capsid assembly.** *JBC* 2006, **281(7):**3773-3784.

63. Sandefur S, Varthakavi V, Spearman P: **The I domain is required for efficient plasma membrane binding of human immunodeficiency virus type 1 Pr55 Gag.** *J Virol* 1998, **72(4):**2723-32.

64. Sandefur S, Smith RM, Varthakavi V, Spearman P: **Mapping and characterization of the N-terminal I domain of human immunodeficiency virus type 1 Pr55 (Gag).** *J Virol* 2000, **74(16):**7238-49.

65. Swanstrom RA and Wills JW: **Synthesis, assembly and processing of viral proteins.** In *Retroviruses* edited by Coffin JM, Hughes SH and Varmus HE. Cold Spring Harbor Laboratory Press, 1997:263-334.

66. Luo K, Liu B, Xiao Z, Yu Y, Yu X, Gorelick R, Yu XF: **Amino-terminal region of the human immunodeficiency virus type 1 nucleocapsid is required for human APOBEC3G packaging.** *J Virol* 2004, **78(21):**11841-52.

67. Krogstad P, Geng YZ, Rey O, Canon J, Ibarrondo FJ, Ackerson B, Patel J, Aldovini A:, **Human immunodeficiency virus nucleocapsid protein polymorphisms modulate the infectivity of RNA packaging mutants.** *Virology* 2002, **294(2):**282-8.

68. Wilk T, Gowen B, Fuller SD: **Actin associates with the nucleocapsid domain of the human immunodeficiency virus Gag polyprotein.** *J Virol* 1999, **73(3):**1931-40.

69. Mirambeau G, Lyonnais S, Coulaud D, Hameau L, Lafosse S, Jeusset J, Justome A, Delain E, Gorelick, RJ, Le Cam E: **Transmission electron microscopy reveals an optimal HIV-1 nucleocapsid aggregation with single-stranded nucleic acids and the mature HIV-1 nucleocapsid protein.** J Mol Biol 2006, **364(3):**496-511.

70. Morellet N, Druillennec S, Lenoir C, Bouaziz S, Roques BP: **Helical structure determined by NMR of the HIV-1 (345-392) Gag sequence, surrounding p2: implications for particle assembly and RNA packaging.** *Protein Sci* 2005, **14(2):**375-86.

71. Roldan A, Russell RS, Marchand B, Gotte M, Liang C, Wainberg MA: **In vitro identification and characterization of an early complex linking HIV-1 genomic RNA recognition and Pr55Gag multimerization.** *J Biol Chem* 2004, **279(38):**39886-94.

72. Accola MA, Hoglund S, Gottlinger HG: **A putative alpha-helical structure which overlaps the capsid-p2 boundary in the human immunodeficiency virus type 1 Gag precursor is crucial for viral particle assembly.** *J Virol* 1998, **72(3):**2072-8.

73. Hill MK, Shehu-Xhilaga M, Crowe SM and Mak J: **Proline residues within spacer peptide p1 are important for Human Immunodeficiency Virus Type 1 infectivity, protein processing, and genomic RNA dimer stability.** *J Virol* 2002, **76(22):**11245-11253.

74. Palmer MT, Kirkman R, Kosloff BR, Eipers PG, Morrow CD: **tRNA Isoacceptor preference prior to retrovirus Gag-pol junction links primer selection and viral translation.** *J Virol* 2007, **81(9):**4397-404.

75. Druillennec S, Caneparo A, de Rocquigny H, Roques BP: **Evidence of Interactions between the Nucleocapsid Protein NCp7 and the Reverse Transcriptase of HIV-1.** *J Biol Chem* 1999, **274(16):**11283-11288.

76. Gurer C, Berthoux L, Luban J: **Covalent modification of human immunodeficiency virus type 1 p6 by SUMO-1.** *J Virol* 2005, **79(2):**910-7.

77. Lee S, Joshi A, Nagashima K, Freed EO, Hurley JH: **Structural basis for viral late-domain binding to Alix.** *Nat Struct Mol Biol* 2007, **14(3):**194-9. Epub 2007 Feb 4.

78. Muller B, Patschinsky T, Krausslich HG: **The late-domain-containing protein p6 is the predominant phosphoprotein of human immunodeficiency virus type 1 particles.** *J Virol* 2002, **76(3):**1015-24.

79. Hemonnot B, Cartier C, Gay B, Rebuffat S, Bardy M, Devaux C, Boyer V, Briant L: **The host cell MAP kinase ERK-2 regulates viral assembly and release by phosphorylating the p6gag protein of HIV-1.** *J Biol Chem* 2004, **279(31):**32426-34.

80. Stuchell MD, Garrus JE, Muller B, Stray KM, Ghaffarian S, McKinnon R, Krausslich HG, Morham SG, Sundquist WI: **The human endosomal sorting complex required for transport ESCRT-I) and its role in HIV-1 budding.** *J Biol Chem* 2004, **279(34):**36059-71.

81. Huang M, Orenstein JM, Martin MA, Freed EO: **p6Gag is required for particle production from full-length human immunodeficiency virus type 1 molecular clones expressing protease.** *J Virol* 1995, **69(11):**6810-8.

82. Garrus JE, von Schwedler UK, Pornillos OW, Morham SG, Zavitz KH, Wang HE, Wettstein DA, Stray KM, Cote M, Rich RL, Myszka DG, Sundquist WI: **Tsg101 and the vacuolar protein sorting pathway are essential for HIV1 budding.** *Cell* 2001, **107(1):**55-65.

83. VerPlank L, Bouamr F, LaGrassa TJ, Agresta B, Kikonhogo A, Leis J, Carter CA: **Tsg101, a homologue of ubiquitin-conjugating (E2) enzymes, binds the L domain in HIV type 1 Pr55 (Gag).** *Proc Natl Acad Sci USA* 2001, **98(14):**7724-7729.

84. Bouamr F, Houck-Loomis BR, De Los Santor M, Casaday RJ, Johnson MC, Goff SP: **The C-terminal portion of the Hrs protein interacts with Tsg101 and interferes with Human Immunodeficiency Virus Type 1 Gag particle production.** *J Virol* 2007, **81(6):** 2909-2922.

85. Ott DE, Coren LV, Copeland TD, Kane BP, Johnson DG, Sowder RC II, Yoshinaka Y, Oroszlan S, Arthur LO and Henderson LE: **Ubiquitin is covalently attached to the p6Gag proteins of human immunodificiency virus type 1 and simian immunodeficiency virus and to the p12Gag protein of Moloney murine leukemia virus.** *J Virol* 1998, **72(4):**2962-2968.

86. Hicke L and Dunn R: **Regulation of membrane protein transport by ubiquitin and ubiquitin-binding proteins.** *Annu Rev Cell Dev Biol* 2003, **19:**141-72.

87. Ott DE, Coren LV, Chertova EN, Gagliardi TD, Schubert U: **Ubiquitination of HIV-1 and MuLV Gag.** *Virology* 2000, **278(1):**111-21.
